# Rational Phase Control in the Synthesis of Cobalt Sulfides

**DOI:** 10.1021/acs.chemmater.4c00911

**Published:** 2024-07-31

**Authors:** Peter H. Edwards, Jeremy R. Bairan Espano, Janet E. Macdonald

**Affiliations:** † Department of Chemistry, Vanderbilt University, Nashville, Tennessee 37235, United States; ‡ Vanderbilt Institute of Nanoscale Science and Engineering, Vanderbilt University, Nashville, Tennessee 37235, United States

## Abstract

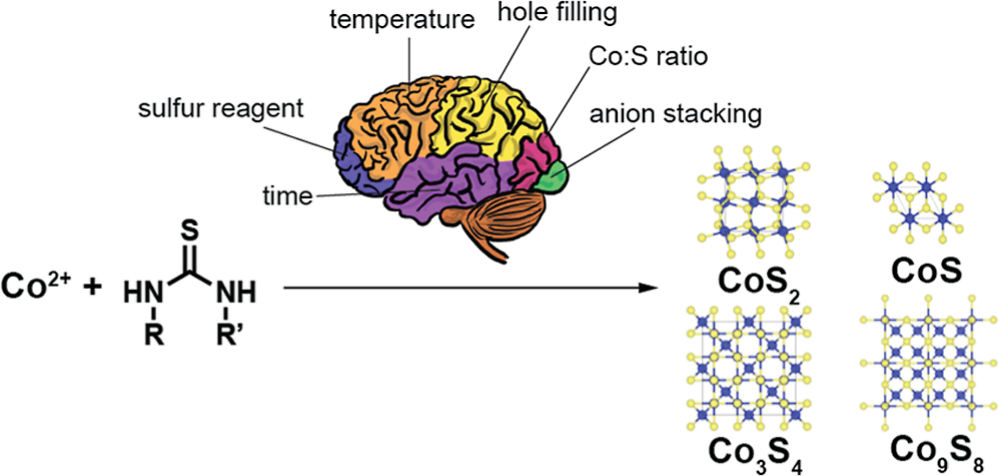

A library of substituted thioureas was used as sulfur reagents in the synthesis of cobalt sulfides. The substitution pattern of the thioureas controls the decomposition rate of precursors into sulfur monomers and thereby aids in the exploration of decomposition kinetics on cobalt sulfide-phase formation, including phase-pure jaipurite (CoS), cobalt pentlandite (Co_8_S_9_), linnaeite (Co_3_S_4_), and cattierite (CoS_2_). We hypothesize that the available transformation pathways between phases during synthesis are dictated by the approximate ccp or hcp stacking of the sulfur lattice. Through gaining a complex understanding of the cobalt sulfide crystal system, phase-pure syntheses of all four naturally occurring crystalline structures in the cobalt sulfide system were achieved.

## Introduction

Transition-metal sulfide nanoparticles have potential applications as batteries,^1−4^ electrocatalysts for clean energy reactions,^5,6^ and treatment methods for human disease.^7^ The cobalt sulfides are prime candidates for material research due to their four naturally occurring crystal phases with well-characterized properties.^8−10^ It has been shown that the changes in phase have a drastic effects on their resulting properties and potential applications. For example it was shown that cattierite (CoS_2_) exhibited superior catalytic performances and long-term stability in the hydrogen and oxygen evolution reaction when compared to linnaeite (Co_3_S_4_) and cobalt pentlandite (Co_9_S_8_).^11^ Linnaeite (Co_3_S_4_) is useful as a stable cathode material in Li/S batteries, while under near-infrared illumination, linnaeite (Co_3_S_4_) causes oxidative modifications and disassociates Alzheimer’s beta-amyloid oligomers. Jaipurite (CoS) has also been reported to have great electrocatalytic activity for oxygen evolution reactions,^12^ as well as great potential as a supercapacitor electrode.^13^ In order to improve these applications, reliable synthetic routes to each of these materials must be established.

There are four naturally occurring cobalt sulfide phases: cattierite (CoS_2_), jaipurite (CoS), linnaeite (Co_3_S_4_), and cobalt pentlandite (Co_9_S_8_) (Table 1).^8,9,14^ Cobalt pentlandite (Co_9_S_8_) has a pseudo ccp sulfur anion packing with 1/2 of the tetrahedral (T_d_) holes filled and 1/8 of the octahedral (O_h_) holes filled with cobalt. Cattierite (CoS_2_) has a pyrite structure with S_2_^2–^ units in ccp packing with octahedral holes filled with cobalt. Linnaeite (Co_3_S_4_) is a spinel, with ccp sulfurs and cobalt in 1/4 of the tetrahedral holes and 1/2 of the octahedral holes. Jaipurite (CoS), the lone hexagonal phase in the cobalt sulfide system; it has a nickel arsenide structure, with hcp sulfurs and all octahedral holes filled with cobalt. There are published syntheses to these phases using either hydrothermal,^15^ colloidal,^4^ solid state,^16^ or vapor deposition approaches.^9^ An unnatural, wurtzite-like phase of CoS (hcp S stacking, with 1/2 of the tetrahedral holes filled by Co) has been synthesized via cation exchange from Cu_2–*x*_S.^17^ Despite these successful approaches, there is a general lack of understanding in the synthetic relationships between each of the cobalt sulfide crystal phases.

**Table 1:** Naturally Occurring Cobalt Sulfides

phase	formula	space group	approximate sulfur lattice	cation hole filling	Δ*H*_f_° (kJ/mol)^8^
cattierite	CoS_2_	*Pa*3̅	S_2_^–2^ ccp	O_h_	–287
jaipurite	CoS	*P*6_3_/*mmc*	S^–2^ hcp	O_h_	–150
linnaeite	Co_3_S_4_	*Fd*3̅*m*	S^–2^ ccp	1/4 T_d_, 1/2 O_h_	–585
cobalt pentlandite	Co_9_S_8_	*Fm*3̅*m*	S^–2^ ccp	1/2 T_d_, 1/8 O_h_	–1326

While there has been some limited success in phase control in hydrothermal synthesis of the cobalt sulfides, there has not been a colloidal study of the synthetic phase control. We must look to studies of other metal sulfides for inspiration. The Brutchey group has pioneered a design of experiments approach to map out the complicated phase space of the copper sulfides using diaryl disulfide precursors.^18^ The van Rohr group has also found success by mapping out the hydrothermal reaction space to achieve marcasite (FeS_2_) over pyrite (FeS_2_).^19^ Our group has found that phase control in iron sulfides is dependent on the chemical reactivity of the sulfur reagents, as measured by C–S bond dissociation energies of organosulfur precursors.^20^ However, direct comparison between the syntheses was problematic because different reagents have unique decomposition mechanisms of sulfur release. Similarly, the Hogarth group found that similar decomposition pathways affected the phase control of nickel sulfides using dithiocarbamate precursors.^21^^1^H NMR revealed that the addition of amine changed the nature of the sulfur precursor in situ. These studies suggest that mapping out the reaction space can give insights into controlling phase, but one must be cognizant of how changing conditions could also change the nature of the reactive precursor.

A set of precursors exhibiting a wide range of decomposition speeds yet similar reaction mechanisms is necessary to understand the role of precursor reactivity on phase control. The Owen group developed a library of thiourea molecules with varying degrees of amine substitution and steric bulk as a means of controlling the rate of sulfur release in the synthesis of lead sulfide nanocrystals. By replacing the hydrogen atoms on nitrogen for various alkyl and aryl groups, the rate of molecule decomposition into free sulfur can be finely controlled over a range of 5 orders of magnitude, offering a unique approach to control nanocrystal size.^22^ This library has also been adapted to study phase-control phenomena to establish a more concrete link between sulfur reagent reactivity and crystal phase. Espano et al. found that the kinetic decomposition speed of the thioureas had a direct impact on phase determination within the iron sulfide crystal system.^23^

When examining the iron-sulfide system more closely, it was found that the initial anion stacking dictated the progression of phases. As nanoparticles nucleated in the most sulfur-deficient, metastable phases, high sulfur concentrations pushed such phases to transform into more sulfur-rich ones. Specifically, cubic mackinawite (FeS) transformed to more thermodynamically stable cubic phases, progressing through a greigite (Fe_2_S_3_) intermediate phase and then pyrite (FeS_2_). The same trend proceeded with the hexagonal phases pyrrhotite (FeS), smythite (Fe_3+*x*_S_4_), and marcasite (FeS_2_).^23^ Using this rationale, six out of the eight iron sulfides were prepared with a high degree of phase purity. The next step is to discover whether the discovered patterns for phase control extend beyond the iron sulfides.^24^ If such trends are found to be generalizable across other transition-metal sulfides, then predictable, algorithmic synthesis methods for many binary compounds may be realized.

The cobalt sulfides make a good test to validate the trends observed in the iron sulfides. The iron sulfides have three sets of naturally occurring polymorphic pairs with approximately hexagonal or cubic close packing of the anions and nearly identical stoichiometries [e.g., cubic pyrite (FeS_2_) and orthorhombic marcasite (FeS_2_)]. However, the cobalt sulfides have only four naturally existing phases and no polymorphic pairs. Many of the stoichiometries, like CoS_2_ or CoS, are only observed naturally in either one of the two symmetries; it is the lack of these polymorphic pairs that make the cobalt sulfides an interesting material of study.

Of the existing phases of cobalt sulfide, jaipurite (CoS) will presumably be the most difficult to synthesize as there are few geologic records in which it is present, and many of the reported nanocrystalline syntheses yielded low nanoparticle crystallinity^25^ or used extreme reaction times and temperatures.^16,26^ Jaipurite is the sole cobalt sulfide phase with approximately hexagonal close packing, and so crystallization must require some unique conditions to favor this arrangement.

Here, we use a library of thioureas to try to understand the overall synthetic landscapes of the cobalt sulfides. By varying the temperature, thiourea-substituent electron-withdrawing character, and the stoichiometric excess of the sulfur precursor, all four natural cobalt sulfides—cattierite (CoS_2_), linnaeite (Co_3_S_4_), cobalt pentlandite (Co_9_S_8_) and, remarkably, jaipurite (CoS)—were synthesized phase pure, thus exhibiting an impressive control over nanocrystalline syntheses. Such results confirm that anion hole stacking and sulfur ratios govern the resulting nanoparticle phases like those of the iron sulfide system.^23^ Specifically, upon nucleation in either a pseudo hexagonal or cubic anion packing, conversion to phases of the alternative symmetry was not observed, but interconversion between phases within each, respective, packing groups was found to occur. There was a strong and direct correlation between the concentration and reactivity of the sulfur precursor and the percent sulfur by mass in the produced phases. Understanding the behaviors of the cobalt sulfide system allows us to rationally synthesize all four cobalt sulfide phases pure in a single set of experiments.

## Methods

### Synthesis of **1**—3,5-Bis(trifluoromethyl)phenyl-3-phenyl-2-thiourea

The synthesis method was adapted from Hendricks et al.^22^ A solution of aniline (6 mmol) in toluene (5 mL) was added to a solution of 3,5-bis(trifluoromethyl) (6 mmol) in toluene (5 mL). The solution was allowed to stir for 5 min. The clear liquid turned white, and the volatiles were removed using vacuum. Characterization:^1^H NMR (CdCl_3_, 400 MHz): 7.34 (d, 2H, *o*-CH (unsub.), 7.41 (t, 1H, *p*-CH (unsub.), 7.52 (t, 2H, *m*-CH (unsub.), 7.69 (s, 1H, *p*-CH (sub.), 7.70 (br, 1H, NH (unsub.), 8.00 (s, 1H, *p*-CH (sub.), 8.29 (br, 1H, NH (sub.).

### Synthesis of 1-Hexyl-3-phenyl-2-thiourea Thiourea

The synthesis method was adapted from Hendricks et al.^22^ (3) A solution of hexylamine (6 mmol) in toluene (5 mL) was added to a solution of phenyl thiocyanate (6 mmol) in toluene (5 mL). The solution was allowed to stir for 5 min. The clear liquid turned white liquid, and the volatiles were removed using vacuum. Characterization: ^1^H NMR (CdCl_3_, 400 MHz): 0.87 (t, 3H, –CH_3_), 1.28 (m, 6H, (CH_2_)_3_), 1.56 (p, 2H, CH_2_), 3.62 (q, 2H, CH_2_), 5.98 (br, 1H, NH), 7.16 (d, 2H, *o*-CH), 7.25 (t, 1H, *p*-CH), 7.43 (t, 2H, *m*-CH), 7.73 (br, 1H, NH).

### General Cobalt Sulfide Nanoparticle Synthesis in 1-Octadecene Using Addition Funnel Hot Addition Method

1 mmol (626 mg) cobalt(II) stearate, 10 mL of 1-octadecene (ODE), and a magnetic stir bar were added to a 25 mL, three-neck round-bottom flask. In a 10 mL glass addition funnel, 6 mmol thiourea was added to 5 mL of ODE, and the funnel was fixed to the round-bottom flask using grease and keck clips. A condenser was connected to the three-neck flask, and the system was attached to a Schlenk line via gas adapter atop the condenser. All openings in the system were capped with rubber septa, and thermocouples were placed through the septa into ODE in both the round-bottom flask and the addition funnel. The system was wrapped in glass wool and heated to 60 °C using a heating mantle and degassed for 30 min under vacuum. The vacuum was then switched to argon gas, and the system was heated to the desired temperature (170, 220, or 270 °C). The contents of the addition funnel were heated gently to 170 °C, and upon heating, thiourea dissolved to yield a faintly yellow, clear solution. The contents of the addition funnel were then added into the round-bottom flask, causing a near immediate color change from blue to black. The solution was left to react for the desired length of time (1 min–2 h). The heating mantle was removed from the system and left to cool to 100 °C, at which point the reaction was quenched with ethanol and then chloroform. These served as the antisolvent and solvent, respectively, used to clean the particles. The nanoparticles were centrifuged at 8700 rpm for 5 min, and the solvents were decanted. This process was repeated twice more before storing the nanoparticles in minimal amounts of chloroform.

### General Cobalt Sulfide Nanoparticle Synthesis Using a One-Pot Method with ODE

1 mmol (626 mg) cobalt(II) stearate, 15 mL of ODE, the desired amount of thiourea, and a magnetic stir bar were added to a 25 mL, three-neck round-bottom flask. A condenser was connected to the three-neck flask, and the system was attached to a Schlenk line via a gas adapter atop the condenser. The two side necks in the system were capped with rubber septa, and a thermocouple was placed through one of the septa and submerged into ODE. The system was wrapped in glass wool and heated to 60 °C using a heating mantle and degassed for 30 min under vacuum. The resulting mixture was light blue with notable solid thiourea crystals. Vacuum was then switched to argon gas, and the system was heated to the desired temperature. The gradual heating caused a color change from blue to black which took place over most of the heating. The solution was left to react for 1 h once the round-bottom reached the desired temperature. After reacting, the heating mantle was removed from the system and left to cool to 100 °C, at which point the reaction was quenched with ethanol and then chloroform. These served as the antisolvent and solvent, respectively, used to clean the particles. The nanoparticles were centrifuged at 8700 rpm for 5 min, and the solvents were decanted. This process was repeated twice more before storing the nanoparticles in minimal amounts of chloroform.

### Cattierite Synthesis

1 mmol (626 mg) cobalt(II) stearate, 10 mL of ODE, and a magnetic stir bar were added to a 25 mL, three-neck round-bottom flask. In a 10 mL glass addition funnel, 12 mmol thiourea (0.913 g) was added to 5 mL of ODE, and the funnel was fixed to the round-bottom flask using grease and keck clips. A condenser is connected to the three-neck flask to account for possible reflux, and the system was attached to a Schlenk line via a gas adapter atop the condenser. All openings in the system were capped with rubber septa, and thermocouples were placed through the septa into ODE in both the round-bottom flask and the addition funnel. The system was wrapped in glass wool and heated to 60 °C using a heating mantle and degassed for 30 min under vacuum. The vacuum was then switched to argon gas, and the system was heated to 220 °C. The contents of the addition funnel were heated gently to 170 °C, and upon heating, thiourea dissolves to yield a faintly yellow, homogeneous solution. The contents of the addition funnel were then quickly added into the round-bottom flask, yielding a near-immediate color change from blue to black. The solution was left to react for 1 h. Once complete, the heating mantle was removed from the system and left to cool to 100 °C, at which point the reaction was quenched with ethanol and then chloroform. These served as the antisolvent and solvent, respectively, used to clean the particles. The nanoparticles were centrifuged at 8700 rpm for 5 min, and the solvents were decanted. This process was repeated twice more before storing the nanoparticles in minimal amounts of chloroform.

### Cobalt Pentlandite Synthesis

1 mmol (626 mg) cobalt(II) stearate, 10 mL of ODE, and a magnetic stir bar were added to a 25 mL, three-neck round-bottom flask. In a 10 mL glass addition funnel, 0.5 mmol 1-hexyl-3-phenyl thiourea (0.119 g) was added to 5 mL of ODE, and the funnel was fixed to the round-bottom flask using grease and keck clips. A condenser was connected to the three-neck flask, and the system was attached to a Schlenk line via a gas adapter atop the condenser. All openings in the system were capped with rubber septa, and thermocouples were placed through the septa into ODE in both the round-bottom flask and the addition funnel. The system was wrapped in glass wool and heated to 60 °C using a heating mantle and degassed for 30 min under vacuum. Vacuum was then switched to argon gas, and the system was heated to 220 °C. The contents of the addition funnel were heated gently to 170 °C, and upon heating, 1-hexyl-3-phenylthiourea dissolves to yield a faintly yellow, homogeneous solution. The contents of the addition funnel were then quickly added into the round-bottom flask, causing a color change from blue to black over the span of several seconds. The solution was left to react for 1 h. Once complete, the heating mantle was removed from the system and left to cool to 100 °C, at which point the reaction was quenched with ethanol and then chloroform. These served as the antisolvent and solvent, respectively, used to clean the particles. The nanoparticles were centrifuged at 8700 rpm for 5 min, and the solvents were decanted. This process was repeated twice more before storing the nanoparticles in minimal amounts of chloroform.

For transmission electron microscopy (TEM), the reaction was left under 220 °C for 20 min instead of 1 h to control the growth of these cobalt pentlandite particles. Once complete, the heating mantle was removed from the system and left to cool to 100 °C, at which point the reaction was quenched with ethanol and then chloroform. These served as the antisolvent and solvent, respectively, used to clean the particles. The nanoparticles were centrifuged at 8700 rpm for 5 min, and the solvents were decanted. This process was repeated twice more before storing the nanoparticles in minimal amounts of chloroform.

### Jaipurite Synthesis

1 mmol (626 mg) cobalt(II) stearate, 15 mL of ODE, 18 mmol diethylthiourea (2.38 g), and a magnetic stir bar were added to a 25 mL, three-neck round-bottom flask. A condenser was connected to the three-neck flask, and the system was attached to a Schlenk line via a gas adapter atop the condenser. The two side necks in the system were capped with rubber septa, and a thermocouple was placed through one of the septa and submerged into ODE. The system was wrapped in glass wool and heated to 60 °C using a heating mantle and degassed for 30 min under vacuum. The resulting mixture was light blue with notable solid diethylthiourea crystals. Vacuum was then switched to argon gas, and the system was heated to 155 °C. The gradual heating initiated a color change from blue to black which took place over most of the heating. The solution was left to react for 1 h once the round-bottom reached 155 °C. After reacting, the heating mantle was removed from the system and left to cool to 100 °C, at which point the reaction was quenched with ethanol and then chloroform. These served as the antisolvent and solvent, respectively, used to clean the particles. The nanoparticles were centrifuged at 8700 rpm for 5 min, and the solvents were decanted. This process was repeated twice more before storing the nanoparticles in minimal amounts of chloroform.

To make more well-defined particles for TEM studies, the Jaipurite was ligated post-reaction by adding 5 mL of oleic acid at 60 °C during the cooling step. The reaction mixture was stirred for 15 min while further cooling. After that, the reaction was quenched with ethanol and then chloroform. These served as the antisolvent and solvent, respectively, used to clean the particles. The nanoparticles were centrifuged at 8700 rpm for 5 min, and the solvents were decanted. This process was repeated twice more before storing the nanoparticles in minimal amounts of chloroform.

### Linnaeite Synthesis

First, phase-pure cobalt pentlandite nanoparticles were made in accordance with the synthesis described above. Once these nanoparticles were made, and their purity was confirmed via X-ray diffraction (XRD), they were resuspended in a minimal amount of chloroform.

Using a pipet, the suspended cobalt pentlandite nanoparticles were transferred into a 2 mL, three-neck round-bottom flask, along with 10 mL of ODE and a magnetic stir bar. In a 1 mL glass addition funnel, 6 mmol diphenylthiourea (1.37 g) was added to 5 mL of ODE, and the funnel was fixed to the round-bottom flask. A condenser was connected to the three-neck flask, and the system was attached to a Schlenk line via a gas adapter atop the condenser. All openings in the system were capped with rubber septa, and thermocouples were placed through the septa into ODE in both the round-bottom flask and the addition funnel. The system was wrapped in glass wool, heated to 100 °C using a heating mantle, and placed under vacuum for 60 min which removed the chloroform and served as a degassing step. Vacuum was then switched to argon gas, and the system was heated to 220 °C. The contents of the addition funnel were heated gently to 170 °C, and upon heating, thiourea dissolved to yield a faintly yellow, homogeneous solution. The contents of the addition funnel were then added into the round-bottom flask. No color change was observed since the original cobalt pentlandite nanoparticle solution was initially black. The solution was left to react for 1 h, after which the heating mantle was removed from the system, and it was left to cool to 100 °C. At this point, the reaction was quenched with ethanol and then chloroform. These served as the antisolvent and solvent, respectively, used to clean the particles. The nanoparticles were centrifuged at 8700 rpm for 5 min, and the solvents were decanted. This process was repeated twice more before storing the nanoparticles in minimal amounts of chloroform.

### Material Characterization

Using a Pasteur pipet, the suspended particles were drop-cast onto a low-background, Si 510 XRD sample plate. Powder XRD measurements were performed with a Rigaku SmartLab powder X-ray diffractometer with a Cu K_α_ (λ = 0.154 nm) radiation source set to 40 kV and 44 mA and a D/teX Ultra 250 1D silicon strip detector. XRD patterns were acquired using a step size of 0.2° at 3°/min. Phase composition of the sample was determined by Rietveld refinement with *R*_wp_ < 10%.

The Rietveld refinement process and particle size calculations were completed using Rigaku PDXL. XRD patterns were obtained using the above characterization method and were compared to reference cards in the JCPDS with the following reference numbers corresponding to the known phases of cobalt sulfide: cattierite—624838; linnaeite—1011056; jaipurite—9008884; and cobalt pentlandite—31753.

TEM was performed on a FEI Tecnai Osiris 200 kV S/TEM. The TEM samples were prepared by dropping a CHCl_3_ suspension of the cobalt sulfide nanocrystals onto a carbon-coated Cu grid.

## Results and Discussion

**Scheme 1:**
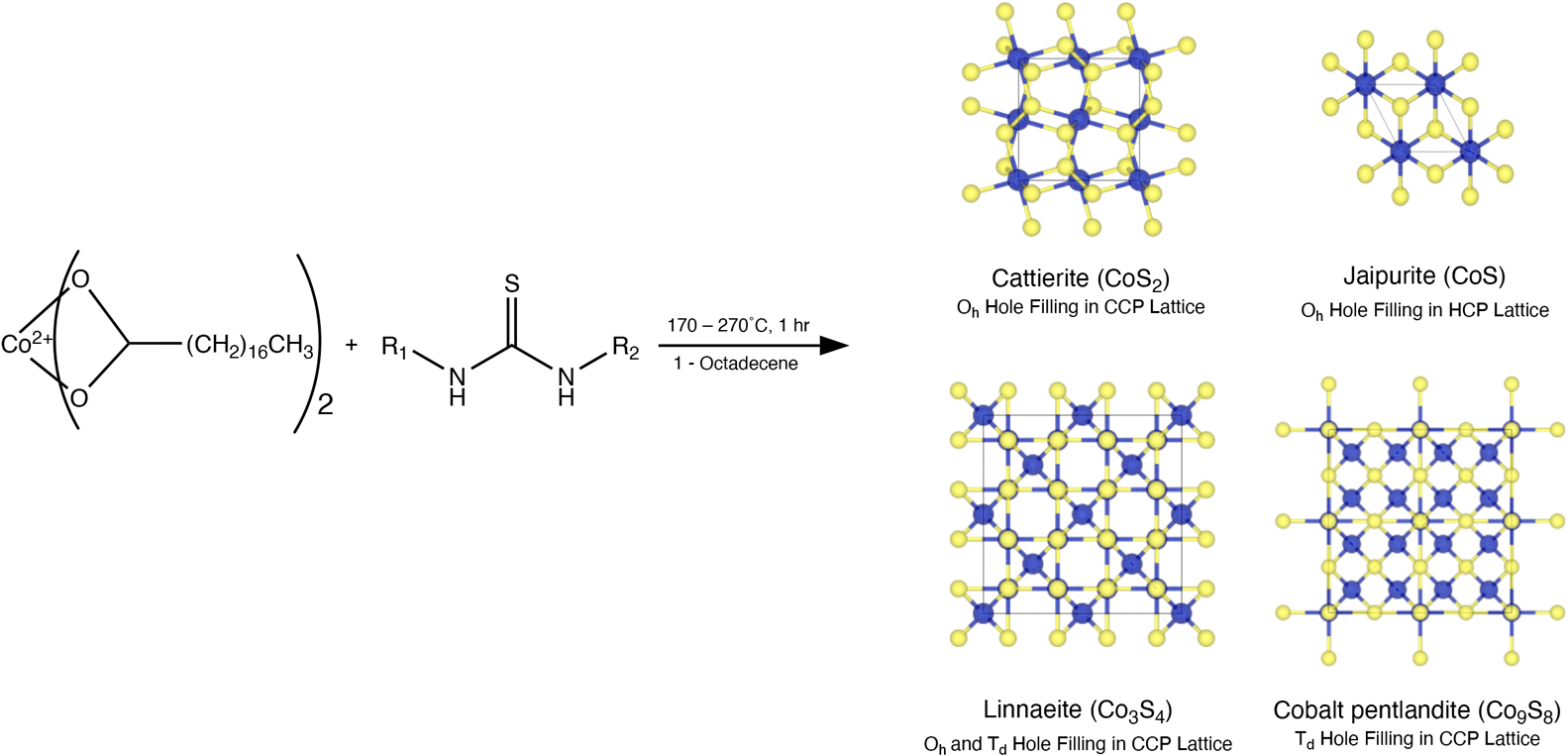
Generalized Synthesis of the Cobalt Sulfides from Cobalt(II) Stearate and Substituted Thioureas

A general synthesis method was developed to study the phase control of cobalt sulfide nanocrystals (Scheme 1). Cobalt(II) stearate was dissolved in ODE at 60 °C and then heated to chosen reaction temperatures of 170, 220, and 270 °C giving a bright blue solution. Separately, six equivalents of the chosen substituted thiourea (thiourea, methylthiourea, phenylthiourea, 1-[3,5-bis(trifluoromethyl)phenyl]-3-phenyl-2-thiourea, and 1,3-diphenylthiourea) were dissolved in ODE in an addition funnel and heated to 170 °C using a heat gun. The ^13^C=S NMR chemical shift of each thiourea was used to indicate of the electron density around the sulfur atom.^22^ The thioureas employed decreased in electron density and reactivity from thiourea > methylthiourea > phenylthiourea > 1-[3,5-bis(trifluoromethyl)phenyl]-3-phenyl-2-thiourea > diphenylthiourea.^23^ The thiourea solution was swiftly added to the vigorously stirring blue cobalt-containing solution, which changed color to black, indicating the formation of cobalt sulfide nanocrystals. The mixture was allowed to react for 1 h. The solutions were cooled to room temperature, and the nanocrystals were isolated by repeated precipitation with ethanol and resuspensions in chloroform. Chloroform suspensions of the products were drop cast on to XRD plates and TEM grids for characterization. When mixtures were present, the composition ratios were determined using Rietveld refinement. It should be noted that powder XRD has a limit of detection of 1–2%.

**Figure 1:**
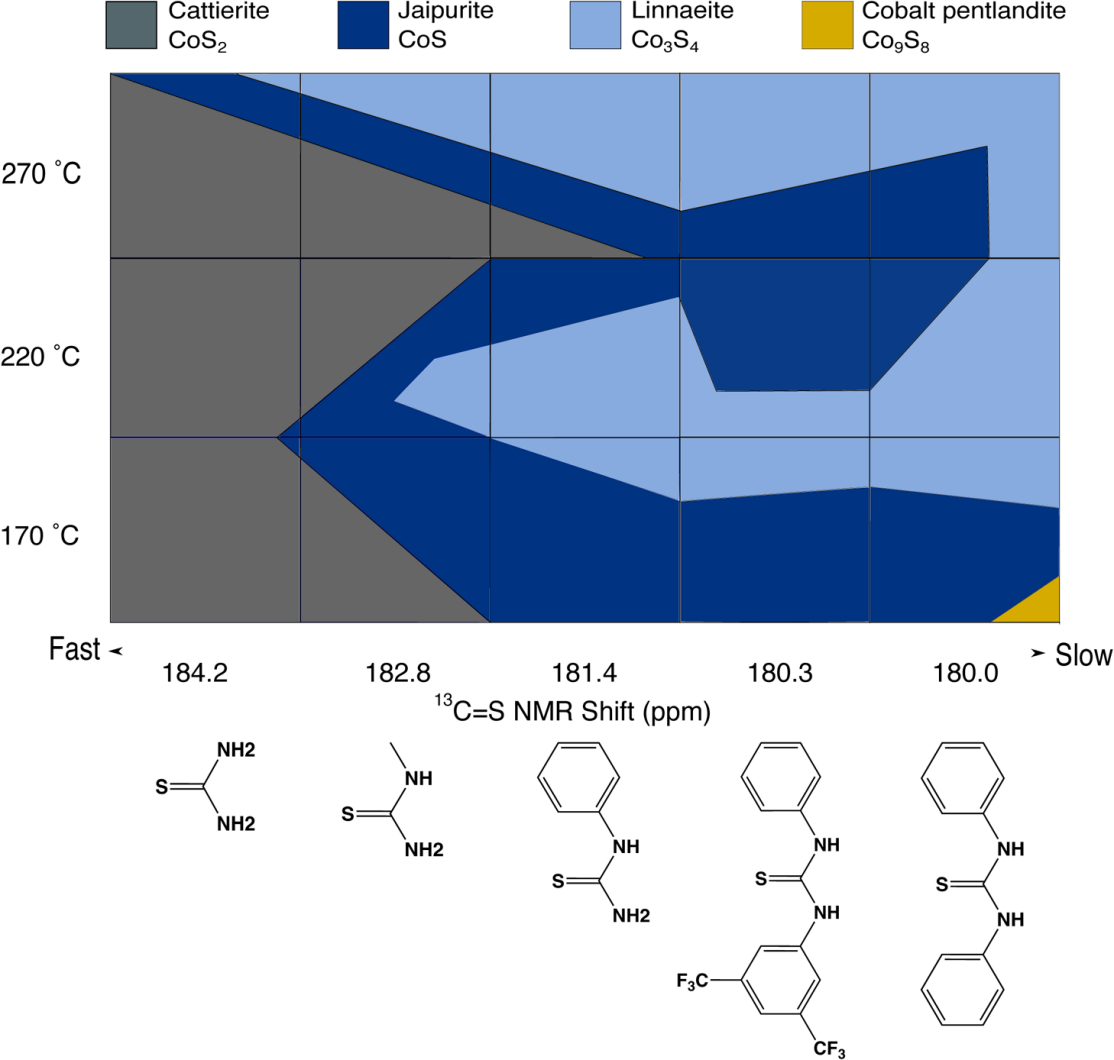
Two-dimensional phase map of cobalt sulfides prepared when cobalt(II) stearate was reacted with substituted thiourea for 1 h. The color of each block indicates the phases present in the product under those conditions, and the areas are the approximate ratio.

A two-dimensional series of reaction were performed controlling reaction temperature and thiourea reactivity. From the diagnostic reactions, each of the four known phases of cobalt sulfide were synthesized as part of mixtures. To help visualize the trends in phase, a synthetic phase map was drawn, showing relative proportions of each phase for each synthetic conditions in the preliminary reactions (Figure 1). Cattierite (CoS_2_) dominates the products for the fastest-reacting thioureas–unsubstituted thiourea and methylthiourea—at all temperatures. Linnaeite (Co_3_S_4_) was the dominant product with slow reacting thioureas—at all temperatures. Jaipurite (CoS) was a minor product in every sample, and a small portion of cobalt pentlandite (Co_9_S_8_) was only seen for the slowest thiourea diphenylthiourea at the lowest synthetic temperature of 170 °C.

A combination of low reaction temperatures and slow thiourea reactivity caused the synthesis of the sulfur-poor phases [jaipurite (CoS), linnaeite (Co_3_S_4_), and cobalt pentlandite (Co_9_S_8_)]. In contrast, when quick-reacting thiourea was employed, regardless of the reaction temperature, the phase mixture contained predominantly the most sulfur-rich phase, cattierite (CoS_2_). The most sulfur-deficient phase cobalt pentlandite (Co_9_S_8_) was only observed in the sample for the slowest-decomposing sulfur precursors at the lowest temperatures. This trend is reminiscent of that identified by Rhodes et al., who found that the sulfur content of specific phases of iron sulfide was inversely related to the strength of the bond dissociation energy or specific organosulfur reagents and thus directly related to the availability of sulfur in the nanocrystal.^20^

The trend is not as simple as “more reactive conditions equals more sulfur in the product”.” While sulfur-rich cattierite (CoS_2_) dominated as a product when reactive thioureas (thiourea and methylthiourea) were employed, at the highest temperature of 270 °C, more sulfur-poor ccp linnaeite (Co_3_S_4_) also formed along with a higher portion of jaipurite (CoS).

A comparison of the enthalpies of formation (Table 1) helps to elucidate some of the trends seen in the initial phase map. Generally, there is an inverse relationship among the cobalt sulfides between their sulfur content and thermodynamic stability. This trend stands in contrast to the iron sulfides, where thermodynamic stability is directly correlated with the amount of sulfur in the mineral’s chemical formula (e.g., FeS_2_ is more thermodynamically stable than FeS),^8,27,28^ in which the most thermodynamically stable cobalt sulfide is cobalt pentlandite (Co_9_S_8_).^8^ In this case, it may be hypothesized that high temperatures allowed the thermodynamic products linnaeite (Co_3_S_4_) and jaipurite (CoS) to form over cattierite (CoS_2_).

**Figure 2:**
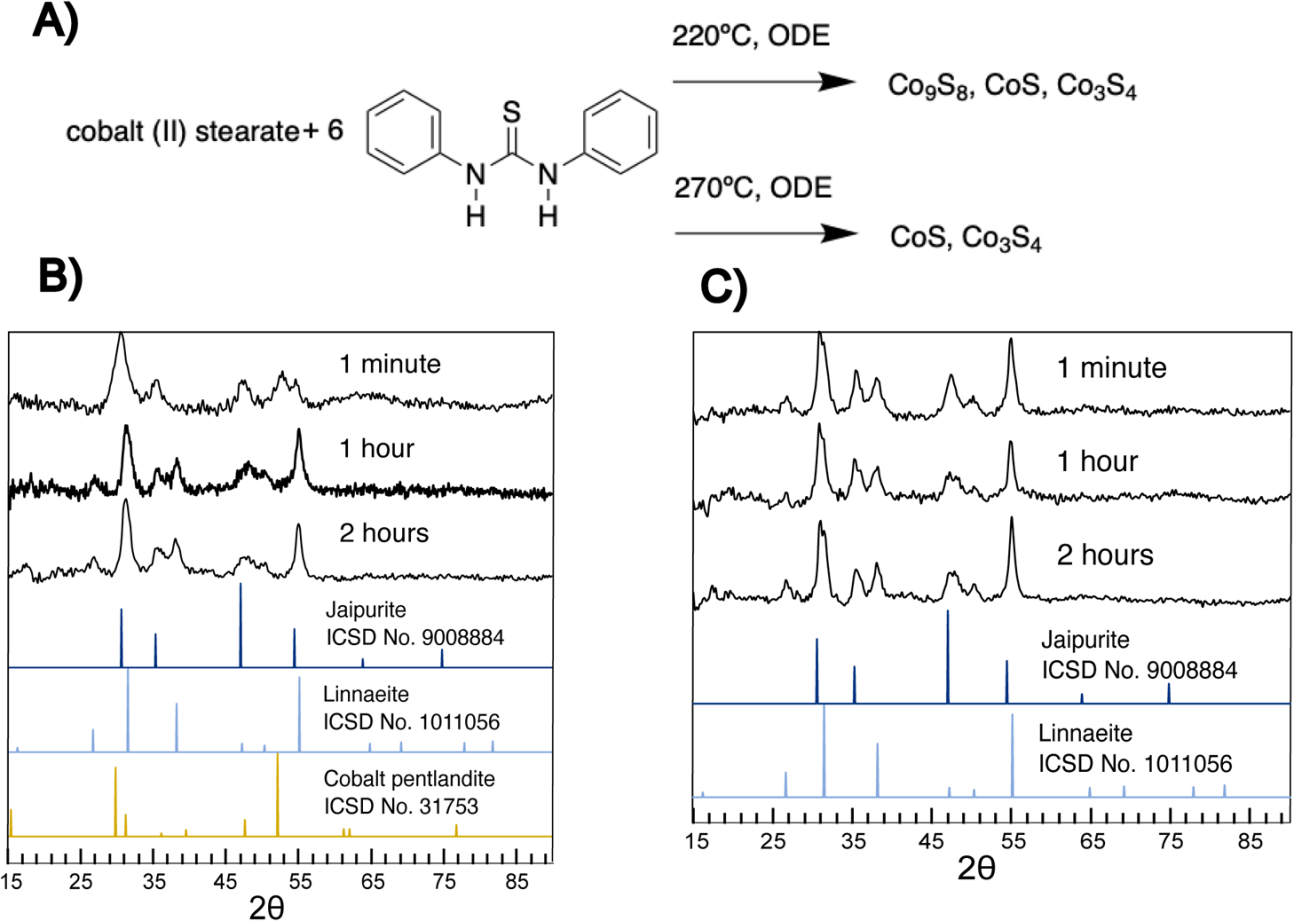
Scheme describing the reaction of cobalt sulfide and diphenyl thiourea in ODE for varying time points, followed by XRD of the products of the synthesis of cobalt sulfides at (a) 220 °C and (b) 270 °C at different time points. In reactions performed at 220 °C, cobalt pentlandite (Co_9_S_8_) transforms into linnaeite (Co_3_S_4_) with time, and the relative portion of jaipurite (CoS) is consistent. At a higher temperature of 270 °C, the products were consistent after 1 min of linnaeite (Co_3_S_4_) and jaipurite (CoS) in the same ratios.

By following reactions of diphenylthiourea with cobalt(II) stearate through time, it was determined that jaipurite is stable and does not transform into another phase, despite being most thermodynamically stable, and cobalt pentlandite (Co_9_S_8_) transforms into linnaeite (Co_3_S_4_) under the excess sulfur conditions (Figure 2). At a temperature of 220 °C, reactions terminated at 1 min gave a product mixture of hcp jaipurite (CoS) and ccp cobalt pentlandite (Co_9_S_8_), with its diagnostic reflection present at 52° 2θ. By 1 h, the reflection at 52° had disappeared, and ccp cobalt pentlandite (Co_9_S_8_) was replaced by the more sulfur-rich ccp linnaeite (Co_3_S_4_), suggesting that cobalt pentlandite (Co_9_S_8_) transforms into linnaeite (Co_3_S_4_) though a diffusive mechanism.^29^ In contrast, at 270 °C, the cobalt pentlandite (Co_9_S_8_) intermediate was not caught, suggesting that the reaction was complete by 1 min at the elevated temperatures. At every reaction point between the 1 min and 2 h time points, there was a consistent presence of hexagonal hcp jaipurite (CoS) in each of the 220 °C samples,^30^ suggesting that once formed, jaipurite is stable under these conditions.

In reactions between diphenylthiourea and cobalt(II) stearate performed at 270 °C, the products were consistently a mixture of linnaeite (Co_3_S_4_) and jaipurite (CoS) for all recorded reaction times. The high temperatures apparently cause diphenylthiourea to decompose into free sulfur so quickly that the reaction is complete by the end of 1 min.

In the past, to understand the iron sulfide phase map, it was necessary to split the phases into those that had approximate cubic close packing (ccp) and hexagonal close packing (hcp) of the sulfurs. The transformations within these groups had nearly independent paths. The same applies here. Jaipurite (CoS) is the only hcp phase of the cobalt sulfides, and the rest is ccp. At different time points (Figure 2), the concentration of hcp jaipurite (CoS) remained consistent, while the ccp phases (linnaeite and cobalt pentlandite) jockeyed for the balance. Furthermore, in the broader phase map (Figure 1) under each set of reaction conditions, there was a mixture of ccp and hcp (i.e., jaipurite) cobalt phases. We hypothesize that hcp and ccp phases cannot easily interconvert (like the iron sulfides). We can note that under all conditions studied thus far, both hcp and ccp nuclei form.

**Figure 3:**
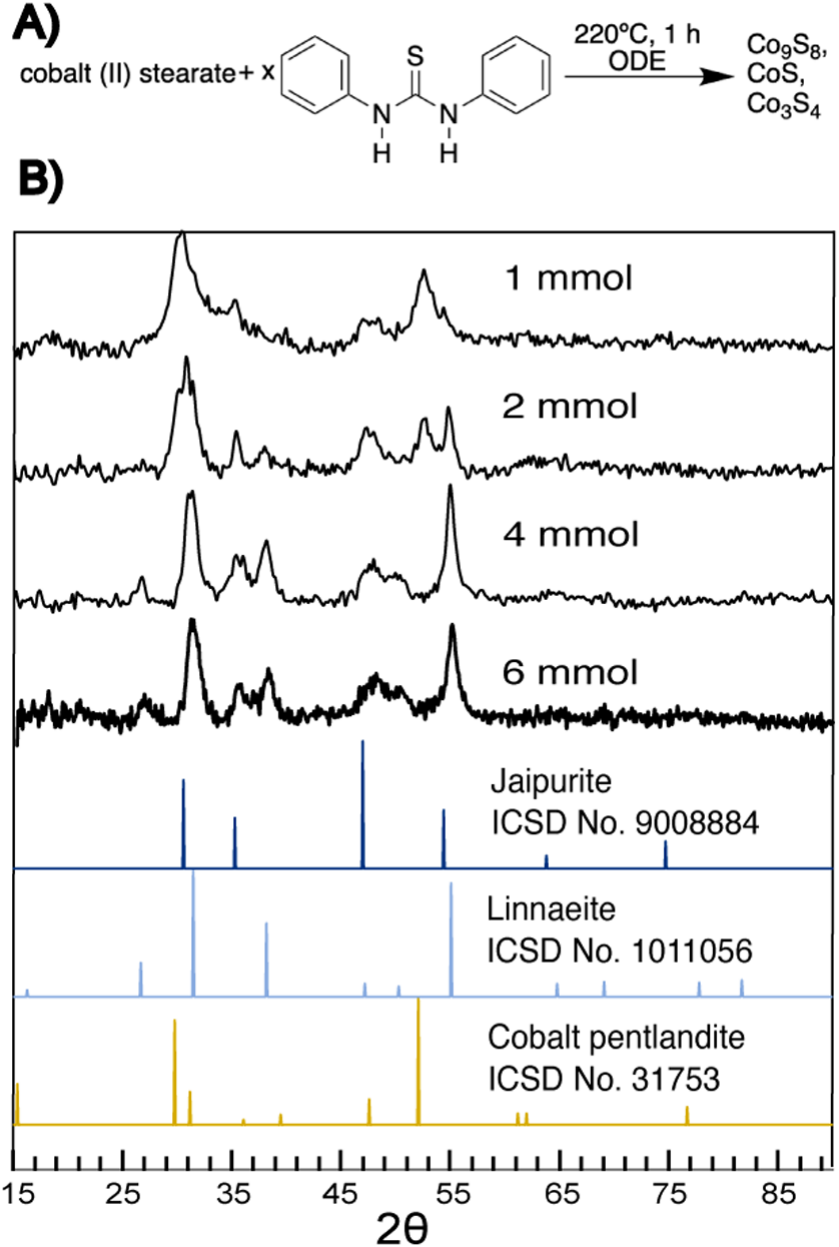
(A) Scheme describing the reactions between cobalt stearate and varying amounts of diphenylthiourea at 220 °C for 1 h, followed by the (B) XRD of the products of the synthesis of cobalt sulfides from cobalt(II) stearate and 1,3- diphenylthiourea at 220 °C.

To understand more on the nucleation of these particles, we explored the effect that sulfur precursor concentration had on phase. We started with the reaction at 220 °C with 1,3-diphenyl thiourea, which resulted in ccp linnaeite (Co_3_S_4_) as the dominant ccp product (Figure 1) when thiourea was in excess 6:1 over cobalt. Lowering the thiourea amount from 6 to 1 eq, the more sulfur-poor ccp cobalt pentlandite (Co_9_S_8_) became increasingly the dominant ccp product over linnaeite (Co_3_S_4_) (Figure 3). The results further suggest that cobalt pentlandite (Co_9_S_8_) is an intermediate to linnaeite (Co_3_S_4_), and precursor stoichiometry is a method to control the relationship.

Surprisingly, in the original 2D phase map, there was no evidence of ccp linnaeite (Co_3_S_4_) further transforming into more sulfur-rich ccp cattierite (CoS_2_). For the fast-reacting thioureas, ccp cattierite (CoS_2_) dominated the product at low temperatures and no ccp linnaeite (Co_3_S_4_) was formed. The more sulfur-poor ccp linnaeite (Co_3_S_4_) was only observed at high temperatures, despite the stoichiometric excess (6:1) of sulfur. These experiments suggests that ccp cattierite (CoS_2_) nucleates directly from solution without sulfur-poor intermediates such as ccp pentlandite (Co_9_S_8_) or ccp linnaeite (Co_3_S_4_). There has only been one study showing the transformation of ccp linnaeite (Co_3_S_4_) to ccp cattierite (CoS_2_), which required oxidizing I_2_ conditions.^31^ The conditions here were reducing as O_2_ was rigorously excluded from the system and the thioureas were was used in excess. While transformation of ccp linnaeite (Co_3_S_4_) into more sulfur-rich ccp cattierite (CoS_2_) is possible, we did not have the correct redox conditions to do so.

From the diagnostic studies, several basic synthetic behaviors can be deduced.

- hcp Jaipurite (CoS) nucleates quickly and independently from the ccp phases under most conditions studied. It does not transform into or from any of the other (ccp) phases under these conditions.
- ccp Cobalt pentlandite (Co_9_S_8_) is an intermediate to ccp linnaeite (Co_3_S_4_) and their ratio in the final product can be influenced by temperature, stoichiometric ratio, and thiourea reactivity.
- ccp Cattierite (CoS_2_) forms under highly reactive sulfur conditions and appears to nucleate directly from solution and does not seem to have an accessible transformation pathway from ccp linnaeite (Co_3_S_4_) or ccp cobalt pentlandite (Co_9_S_8_) under these conditions.

With these observations in hand, we can now rationally seek conditions that target phase-pure (within the limit of detection of XRD) products of each of the cobalt sulfides.

### Phase-Pure Cattierite

Cattierite (CoS_2_) is the dominant product when fast-reacting thioureas are employed but is always accompanied by small amounts of jaipurite (CoS) (Figure 1) and is consistent with the notion that there is apparent conucleation of ccp and hcp phases. However, we can take advantage of the large differences in sulfur content between the two phases to promote the formation of CoS_2_ over CoS; based on the high sulfur content of cattierite (CoS_2_), and with the large occurrence of the phase with fast-reactive thioureas, we hypothesized that high concentrations of fast-reactive thioureas would be selected for this phase.

**Figure 4:**
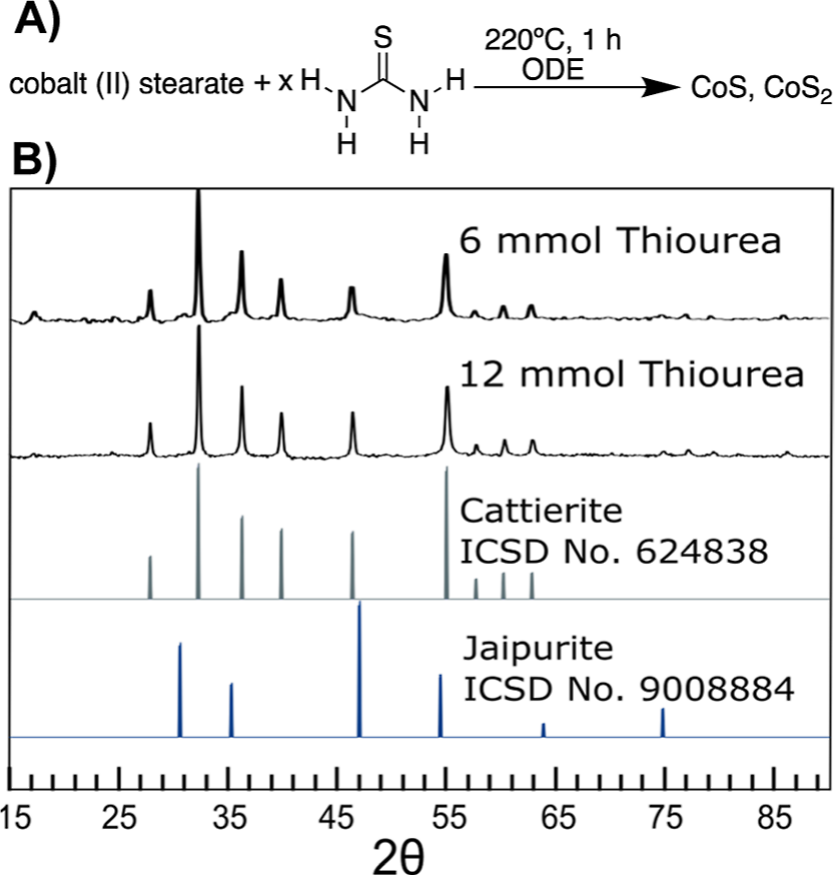
XRD of the products of the synthesis of cobalt sulfides from cobalt(II) stearate and thiourea at 220 °C.

Further experimentation found that by doubling the amount of thiourea from 6 to 12 equivalents of the sulfur precursor per equivalent of the cobalt precursor, the amount of jaipurite (CoS) fell below the detection limit of the XRD. Jaipurite (CoS) can often be observed as low-angle shoulders to the reflections at 33 and 36° in XRD; these shoulders were not present, suggesting that cattierite (CoS_2_) was the only detected product (Figure 4).

**Figure 5:**
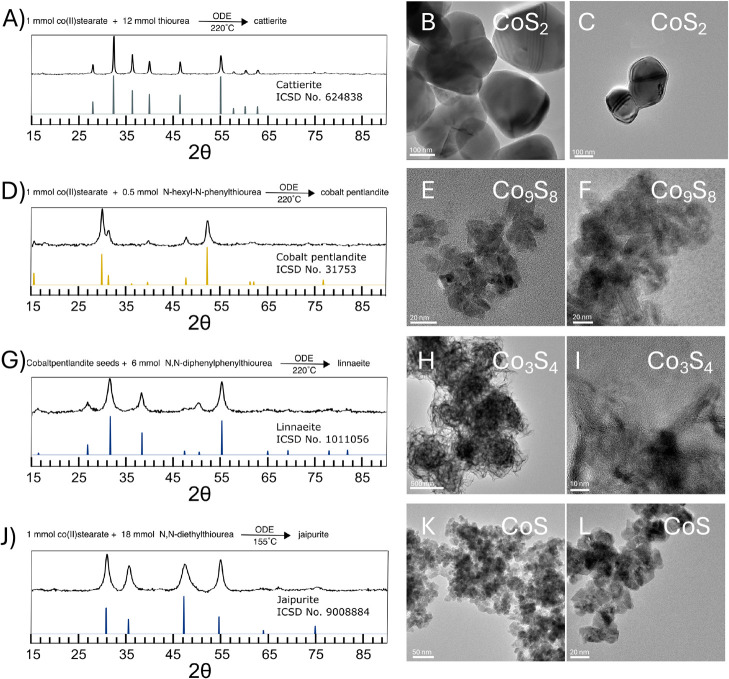
Phase-pure colloidal syntheses to (A–C) cattierite (CoS_2_), (D–F) cobalt pentlandite (Co_8_S_9_) (G–I), linnaeite (Co_3_S_4_), and (J–L) jaipurite (CoS) accompanied by their corresponding XRD patterns and TEM images. For the phase-pure jaipurite synthesis TEM images, the reaction was adapted to help ligate the nanocrystals by adding 5 mL of oleic acid to the solution post reaction.

In the optimized reaction (Figure 5A–C), TEM of the cattierite (CoS_2_) product showed >100 nm irregularly shaped particles. Every particle showed regions of differing contrast or Moiré patterns, indicating polycrystalline nature. Scherrer line broadening analysis of the XRD pattern yielded a crystallite size of 60 nm, which further suggests that most or all particles are polycrystalline. Figure 5B includes a particle with multiple wedge-shaped crystallites. We can hypothesize that direct nucleation of cattierite starts from a cluster that promotes independent crystallite growth in multiple directions, and identifying and understanding this event more deeply are a potential direction for further research.

### Phase- Pure Cobalt Pentlandite

**Figure 6:**
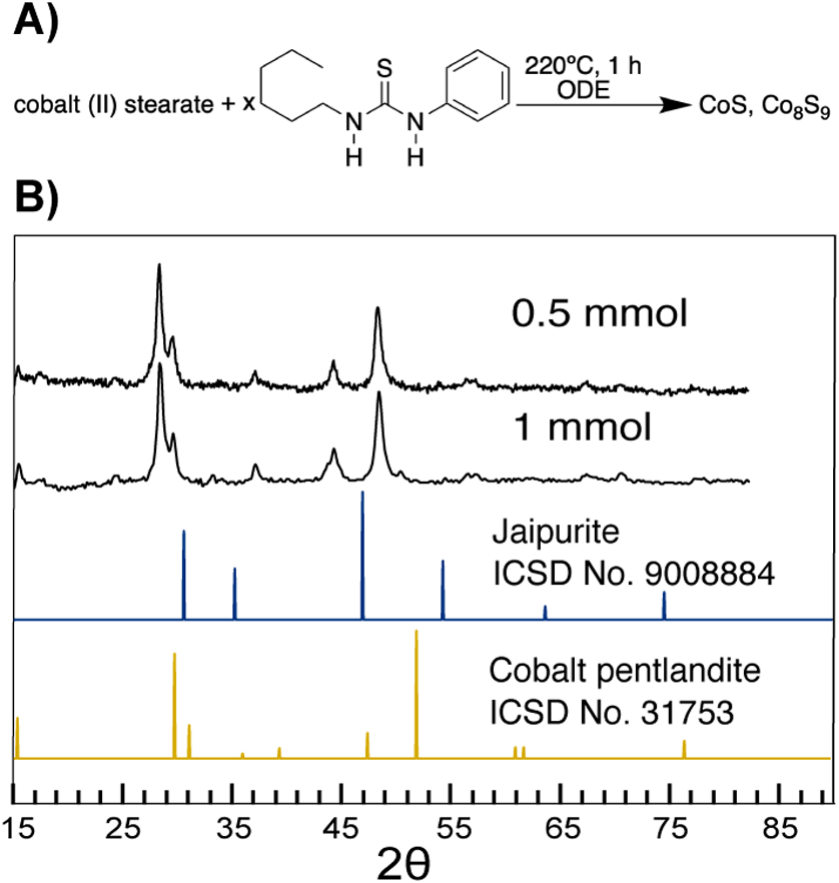
(A) Scheme describing the phase-pure synthesis of cobalt pentlandite (Co_9_S_8_) by using hexylphenylthiourea as the sulfur source, 220 °C, and lowering the Co/S ratio to 0.5:1, followed by the (B) corresponding XRD patterns. At higher ratios, impurities of the more sulfur-rich linnaeite (Co_3_S_4_) and jaipurite (CoS) were present.

Cobalt pentlandite (Co_9_S_8_) is the most sulfur poor of all the cobalt sulfides. It is interesting to note that despite cobalt pentlandite (Co_9_S_8_) being the most thermodynamically stable phase of the cobalt sulfides with a Δ*H*_f_° = −1326 kJ/mol,^8^ this was the least-abundant phase found in the phase map, likely because excess sulfur precursors were employed in initial studies. Cobalt pentlandite (Co_9_S_8_) crystals were only observed in substantial quantities in syntheses which used low sulfur precursor reactivity (diethyl thiourea and diphenylthiourea) at the lowest temperature of 170 °C. We can conclude that the phase map is driven not by thermodynamic stabilities of the phases but more so by the chemical potential of the sulfur environment. Thus, using slow reactive thioureas would enable us to synthesize cobalt pentlandite (Co_9_S_8_), and decreasing the thiourea concentration would help to only nucleate that phase. Also considering that cobalt pentlandite (Co_9_S_8_) has some of the cobalt atoms in T_d_ coordination, while they are entirely octahedral in jaipurite (CoS), low sulfur availability could favor low coordination structures. Thus, we optimized the cobalt pentlandite (Co_9_S_8_) synthesis by using a combination of slow-reactive thioureas and low sulfur precursor concentrations (Figure 6).

In the initial experiments, cobalt pentlandite (Co_9_S_8_) was observed in mixtures with linnaeite (Co_3_S_4_) and jaipurite (CoS) (Figure 1) when using slowly reacting disubstituted thioureas at low temperatures, which could be attributed to cobalt pentlandite’s (Co_9_S_8_) low-sulfur content. Lowering the sulfur to cobalt ratio and stoichiometric ratios from 6:1 to 1:1 at 220 °C prevented the transformation of sulfur-poor cobalt pentlandite (Figure 3) to linnaeite (Co_3_S_4_) and resulted in an enhancement of the former in the product. At a 1:1 sulfur to cobalt ratio in solution, the cobalt pentlandite (Co_9_S_8_) was completely free of linnaeite (Co_3_S_4_), and only a small impurity of the slightly more sulfur-rich hcp jaipurite (CoS), as seen by a characteristic XRD shoulder at 46° (Figure 6). Further lowering the ratio to 0.5:1 removed the Jaipurite impurity, suggesting that sulfur-deficient conditions with low-sulfur chemical potential prevents the nucleation of the hcp stacking, in favor of ccp stacking.

TEM of the optimized reaction (Figure 5D–F) showed aggregates of 10–15 nm crystals. Aggregation is to be expected since these reactions were performed without the additional variable of the ligand. Scherrer line broadening of the XRD pattern gave a crystallite size of 12 nm, suggesting that particles are single crystalline.

### Phase-Pure Linnaeite

The synthesis of phase-pure hcp jaipurite (CoS) and ccp linnaeite (Co_3_S_4_) presents a unique challenge. Since linnaeite (Co_3_S_4_) and jaipurite (CoS) both have intermediate sulfur contents when compared to cattierite (CoS_2_) and cobalt pentlandite (Co_9_S_8_), forcing sulfur concentrations could not be used as a direct synthetic handle. In the initial experiments, we hypothesized that ccp cobalt pentlandite was an intermediate to ccp linnaeite (Co_3_S_4_) and could be potentially accessed by performing a seeded synthesis using sulfur-poor cobalt pentlandite (Co_9_S_8_) seeds and injecting additional thiourea.^8,14^ This seeded growth approach was successful in the iron sulfides; pyrrhotite (Fe_7_S_8_) was used as hcp seeds and reacted with a medium-reactive thiourea to produce hcp marcasite (FeS_2_).^23^

Therefore, phase-pure linnaeite (Co_3_S_4_) was achieved through a diffusive phase transformation between cobalt pentlandite (Co_9_S_8_) and linnaeite (Co_3_S_4_) in a two-step procedure. Cobalt pentlandite (Co_9_S_8_) nanoparticles were synthesized and cleaned three times using a solvent and antisolvent of chloroform and ethanol, respectively. The seeds then reacted with 6 mmol of slowly reacting diphenylthiourea to force excess sulfur into the cubic framework of cobalt pentlandite (Co_9_S_8_) to yield linnaeite (Co_3_S_4_) (Figure 5G). This method was successful in bypassing the observed conucleation of hexagonal jaipurite (CoS) since there is no known path of interconversion between the hcp and ccp families of phases in the cobalt sulfides under these reducing conditions.

TEM of the linnaeite (Co_3_S_4_) showed agglomerated sheet-like structures (Figure 5E,F). Greigite (Fe_3_S_4_) has a similar cubic spinel structure which also is known to give sheet-like structures from colloidal synthesis.^32^

### Synthesizing Phase-Pure Jaipurite

Phase pure jaipurite (CoS) is the most difficult to synthesize due to it being the most metastable of the cobalt sulfides and having an intermediate sulfur stoichiometry.^8,9^ It is the only phase to have hcp sulfurs, and there are no known colloidal syntheses to phase-pure jaipurite (CoS).

Jaipurite (CoS) was targeted by starting with conditions that give a mixture with cobalt pentlandite (Co_9_S_8_) and jaipurite (CoS) and then rationally tweaking the conditions to exploit their differences. First, jaipurite (CoS) has octahedral coordination of the cobalt, whereas cobalt pentlandite (Co_9_S_8_) has predominantly tetrahedral coordination, thus an excess of the sulfur precursor will encourage the more higher coordinate crystal structure. Finally, the presence of hcp jaipurite (CoS) as in impurity phase in almost all syntheses, despite being the most metastable, suggests that it must be the preferred phase to nucleate over cubic cobalt pentlandite (Co_9_S_8_). Therefore, these two ideas will enable us to target one phase over the other.

**Figure 7:**
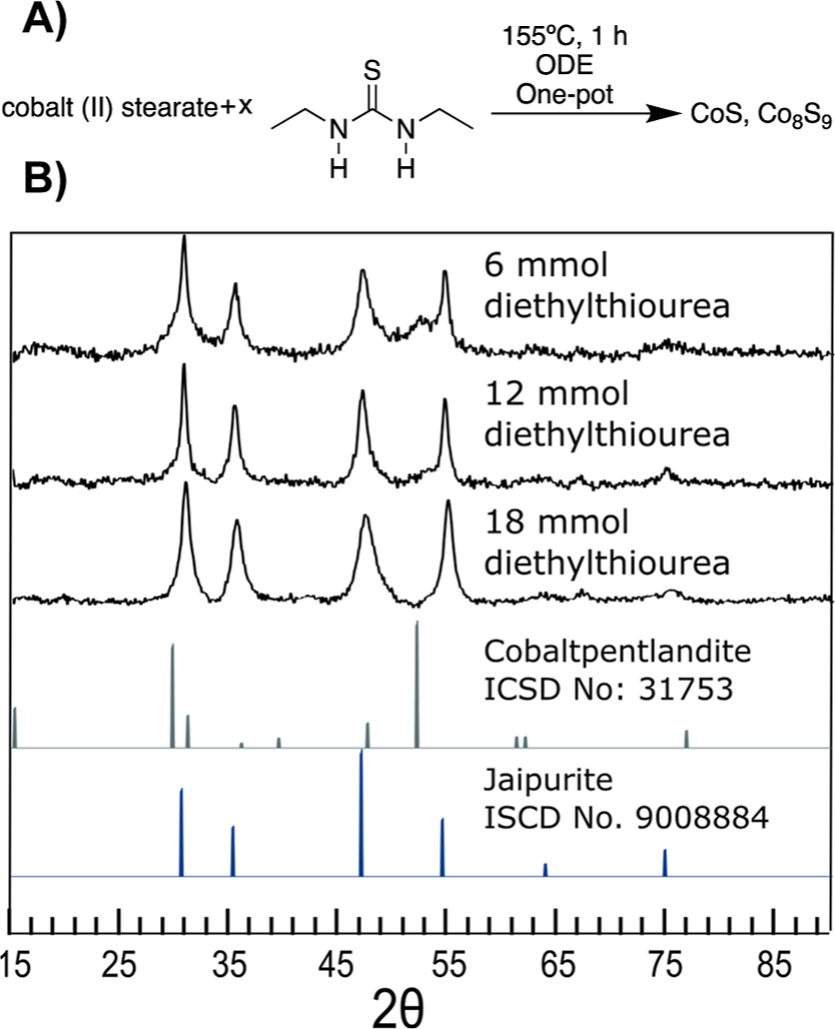
Products of the reactions using variable concentrations of diethylthiourea in a one-pot synthesis method in the pursuit of phase-pure jaipurite. All reactions occurred at 155 °C for 1 h, and it was seen that with increasing diethylthiourea concentration, the amount of cobalt pentlandite approached the detection limit of XRD.

We began with using the reaction conditions of diethyl thiourea at a low temperature of 155 °C which was known to give a mixture of jaipurite (CoS) and cobalt pentlandite (Co_9_S_8_). Instead of a hot injection, a one-pot method in which the cobalt and sulfur precursors were degassed and heated to the reaction temperature in the same vessel was employed. The aim was to cause nucleation at the very minimum necessary temperature to lead to preferential hcp nucleation. A series of reactions were performed in which the amount of the sulfur precursor was varied within this one-pot reaction scheme (Figure 7). It was found that a vast excess of sulfur favored jaipurite (CoS), with an octahedral coordination around Co. In the optimized reaction, phase-pure jaipurite (CoS) was the product of a heat-up one-pot reaction to a temperature of 155 °C with 18 equiv of diethylthiourea (Figure 5J). No impurities of cobalt pentlandite (Co_9_S_8_) were seen within the detection limit of the XRD experiment. Here, by flooding the system with slow reactive thioureas, we prevent the nucleation of cattierite (CoS_2_) and simultaneously push for octahedral hole filing phases to predominate the mixture. When preparing a sample for TEM, oleic acid was added after the reaction was completed to the reaction at ∼60 °C to aid with suspension through the cleaning process. The product was imaged as aggregates of 5–15 nm crystallites (Figure 5J–L), and Scherrer line broadening analysis of the XRD pattern similarly suggested a crystallite size of 9 nm.

## Conclusions

Using various thiourea precursors with different degrees of organic substitution, it was determined that the rate of decomposition of these molecules into a sulfur monomer has a significant effect on phase control phenomena among other transition-metal sulfides, particularly cobalt sulfide. By manipulating the reactivity of specially designed sulfur precursors, each of the known phases of cobalt sulfide were identified in synthesis products and ultimately isolated via phase-pure-direct synthesis; a feat which has yet to be observed when using a single synthetic systematic method. The rate of decomposition was closely related to the resulting cobalt sulfide phase, with the high rate of sulfur release from more-reactive sulfur precursors contributing to the nucleation and growth of more sulfur-rich phases. Unsubstituted thiourea reacts with cobalt to yield cattierite (CoS_2_), especially at lower temperatures, and decreasing the rate of decomposition yields mixtures of phases with a lower proportion of sulfur, such as linnaeite (Co_3_S_4_), cobalt pentlandite (Co_9_S_8_), and jaipurite (CoS). This observation lead to a high level of synthetic control of crystal phase, especially when combined with other observed trends which relate thermodynamic stability and sulfur content of the individual phases to variables like temperature, precursor concentration, and synthesis method.

In comparison to our previous work with the iron sulfides, the cobalt sulfides both confirm these studies and build upon them in a new way. The iron sulfides could be controlled by considering only the anion-stacking pattern and the sulfur content. For the cobalt sulfides, anion stacking was important; however, considering the coordination number around the cobalt was also important to success for the synthesis of jaipurite (CoS). The iron sulfides exhibited a single synthetically accessible path between the hcp and ccp phases at a stoichiometry of approximately FeS,^23^ whereas the cobalt sulfides showed no similar transformation.

Using this approach is valuable not only for further investigation into the cobalt sulfides and their interactions with various ligands and solvents but also for the development of additional synthetic paths to transition-metal chalcogenide nanoparticles.^31^ This study, in combination with the previous work on the iron sulfides, can also be used to understand the phase relationships in mixed metal systems and how chosen binary and ternary metal systems may be synthesized for catalytic or magnetic applications. Beyond synthetic chemistry, these studies may provide insights into geochemical processes. Many metal sulfide minerals are mined doped with other metals, especially among the iron, cobalt, and nickel sulfides, and mysteries remain concerning their formation and phase selection. For example, a disconnect remains in understanding how pyrrhotite (FeS) is doped with cobalt through continuous stoichiometries to give the structurally related jaipurite (CoS) (both are based on the NiAs structure) or under some conditions cobalt pentlandite (Co_9_S_8_) structures can only support a limited ratio of iron.^33^

## Supporting Information Available

Detailed descriptions of the synthetic procedures, XRD patterns of ratio and time studies, Rietveld refinement percentages, and Rietveld refinement XRD patterns (PDF)

## Abbreviations

ODE: CC octadecene
XRD: X-ray diffraction
ccp: CC cubic closed pack
hcp: CC hexagonal closed packed
