# Supplementary material for: Rational Phase Control in the Synthesis of Cobalt Sulfides
Source: Chem Mater. 2024 Jul 31;36(15):7186–96. doi: 10.1021/acs.chemmater.4c00911 (PMC11325534; doi:10.1021/acs.chemmater.4c00911)
Supplement: Supplementary file 1 — cm4c00911_si_001.pdf [file cm4c00911_si_001.pdf]

Rational Phase Control in the Synthesis of Cobalt Sulfides

Peter H. Edwards,[1] Jeremy R. Bairan Espano,[2] and Janet. E. Macdonald*[1,2]

[1]Department of Chemistry and [2]Interdisciplinary Materials Science Program, Vanderbilt University, Nashville, Tennessee 37235, United States

Table of contents

Materials 2

Linnaeite Synthesis 2

Diethylthiourea Timed Trials 3

XRD's for Synthetic Phase Map 4

Rietveld Refinements 5

References 20

Materials

Chemicals. 1-Octadecene (C18H36, 90%,), thiourea (99%), methyl thiourea (97%), phenyl thiourea (98%), diphenyl thiourea (98%), 3,5- bis(trifluoromethyl)phenyl isothiocyanate (98%), phenyl thiocyanate (99%), hexylamine (98%), aniline (99.5), toluene (HPLC grade) was purchased from Sigma Aldrich. Cobalt (II) stearate was purchased from Strem.

Synthesis of Linnaeite

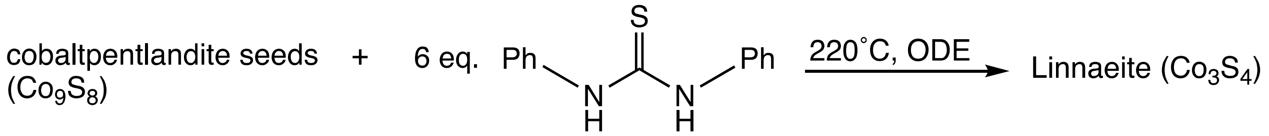

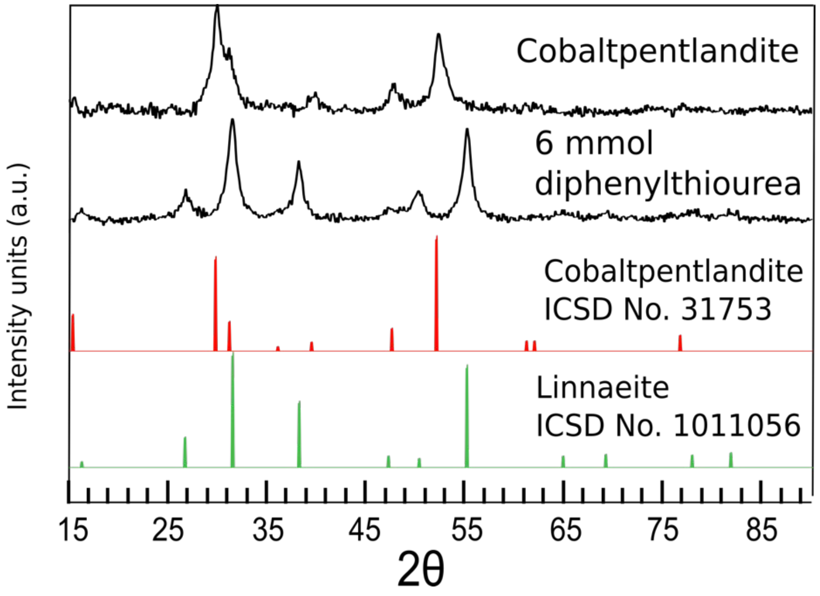

Figure S1: XRD of syntheses which must be performed to yield pure linnaeite. By first synthesizing pure cobaltpentlandite via the procedure above, this can then be reacted in the presence of an excess (6 mmol) of diphenylthiourea to yield linnaeite

Diethylthiourea Ratio Trials

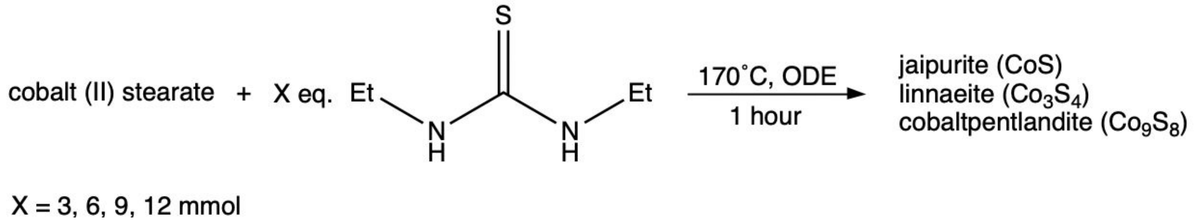

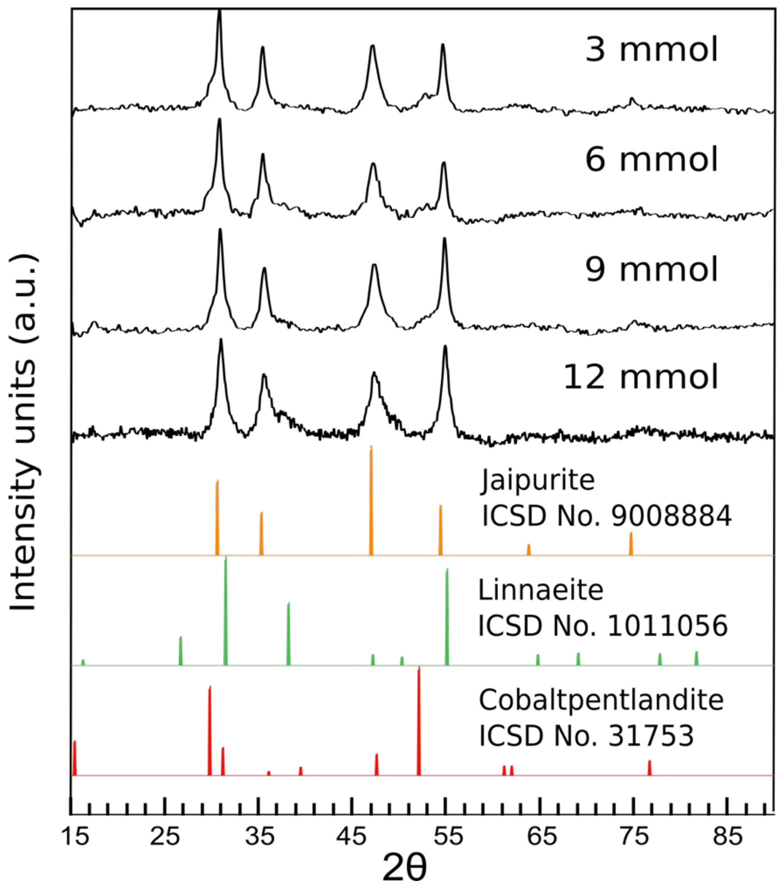

Figure S2: XRD patterns of syntheses performed at 170˚C via a hot addition method using diethylthiourea. Syntheses were performed using various ratios of diethylthiourea. It was determined 9mmol diethylthiourea was the optimal ratio for synthesizing phase pure jaipurite because it yielded a minimal amount of cobaltpentlandite and linnaeite

Using diethylthiourea as the sulfur precursor, a series of experiments were performed in which the starting concentration was varied from 3mmol to 12 mmol. Such experiments showed trends that were seen in other sets of syntheses. At low concentrations of diethylthiourea, there was a higher proportion of the sulfur-poor cobaltpentlandite, as noted by the greater intensity of the peak seen 52˚. With increasing diethylthiourea concentration, the proportion of

cobaltpentlandite decreased as the excess sulfur within the reaction led to the formation of a more sulfur rich phase in jaipurite. However, at the 12 mmol concentration, this amount of sulfur exceeded the minimum concentration of diethylthiourea necessary to synthesize linnaeite. This was because the large excess of sulfur pushed the transformation of cobaltpentlandite nanoparticles into more sulfur-rich linnaeite.

<u>Diethylthiourea Time Trials</u>

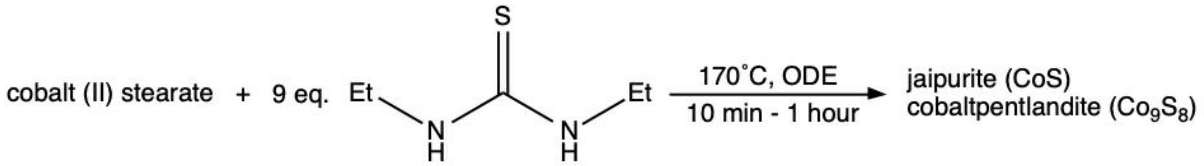

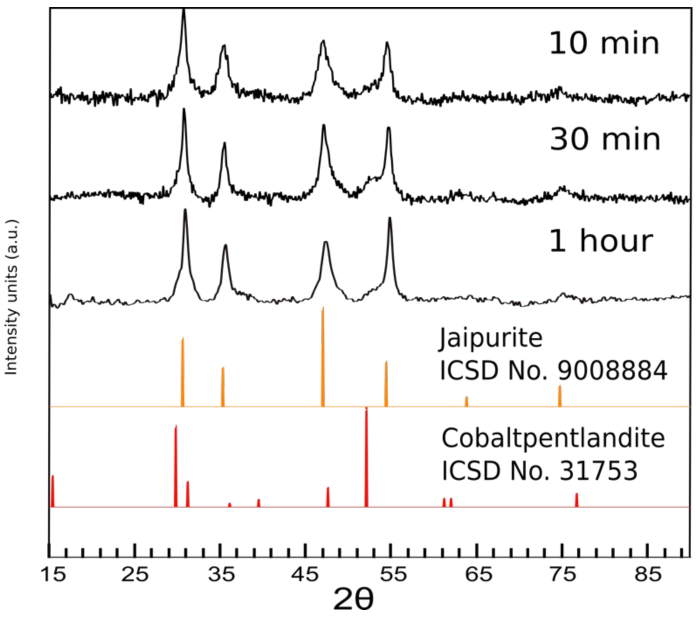

Figure S3: Time trials using 9 mmol of diethylthiourea under the same conditions as experiments performed in Figure S2. Varying the reaction time impacted the crystallinity but failed to yield larger changes in the phase composition of the reaction products. Rietveld refinements for reactions shown in Figure S4.

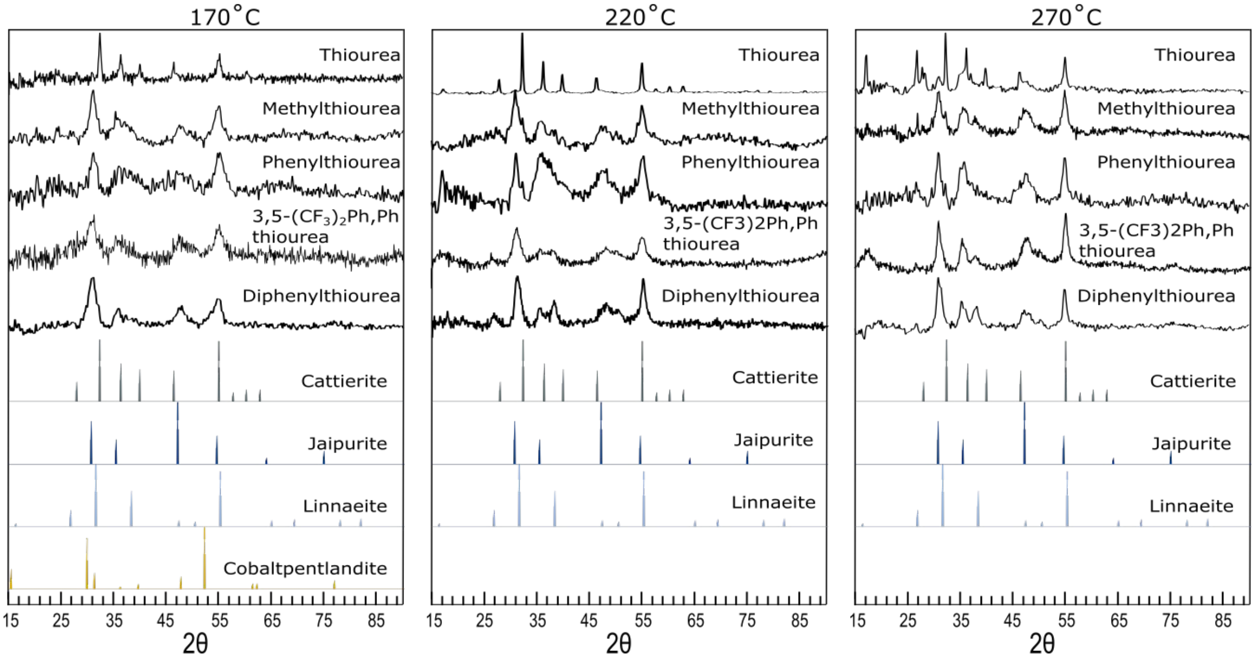

Figure S4: XRD from reactions of cobalt sulfides using substituted thioureas as sulfur reagents at synthetic temperatures of 170ºC, 220ºC, and 270ºC that are represented in the bottom-up synthetic phase map.

Rietveld Refinements

Table S1. Summary of Refinements

	Solvent	T(°C)	Thiourea	S:Ni	Time	Catteirite (CoS_2) 624838	Linnaeite (Co_3S_4) 1011056	Cobaltpentlandite (Co_9S_8) 31753	Jaipurite (CoS) 9008884
1	ODE	170	Thiourea	6 to 1	1 h	98%			2%
2	ODE	170	Methyl Thiourea	6 to 1	1 h	61%			39%
3	ODE	170	Phenyl Thiourea	6 to 1	1 h	27%	53%		19%
4	ODE	170	1-[3,5-(CF_3)Ph]-3-Ph-Thiourea	6 to 1	1 h		45%		55%
5	ODE	170	Diphenyl Thiourea	6 to 1	1 h		58%	10%	32%
6	ODE	220	Thiourea	6 to 1	1 h	98%			2%
7	ODE	220	Methyl Thiourea	6 to 1	1 h	34%	37%		29%
8	ODE	220	Phenyl Thiourea	6 to 1	1 h		39%		61%
9	ODE	220	1-[3,5-(CF_3)Ph]-3-Ph-Thiourea	6 to 1	1 h		30%		70%
s	ODE	220	Diphenyl Thiourea	6 to 1	1 h		47%		53%
11	ODE	270	Thiourea	6 to 1	1 h	66%			34%
12	ODE	270	Methyl Thiourea	6 to 1	1 h	30%	31%		39%
13	ODE	270	Phenyl Thiourea	6 to 1	1 h		55%		45%
14	ODE	270	1-[3,5-(CF_3)Ph]-3-Ph-Thiourea	6 to 1	1 h		40%		60%
15	ODE	270	Diphenyl Thiourea	6 to 1	1 h		70%		30%
16	ODE	220	Thiourea	12 to 1	1 h	100%			
17	ODE	220	1-Hexyl-3-Phenyl Thiourea followed by Diphenyl Thiourea	0.5 to 1 Followed by to 6 to 1	1 h		100%		
18	ODE	220	1-Hexyl-3-Phenyl Thiourea	0.5 to 1	1 h			100%	
19	ODE	155	Diethyl Thiourea	18 to 1	1 h				100%
20	ODE	220	Diphenyl Thiourea	6 to 1	1 min			75%	25%
21	ODE	220	Diphenyl Thiourea	6 to 1	2 h		47%		53%
22	ODE	270	Diphenyl Thioruea	6 to 1	1 min		59%		41%
23	ODE	270	Diphenyl Thiourea	6 to 1	2 h		53%		47%
24	ODE	220	Diphenyl Thiourea	1 to 1	1 h		33%	67%	
25	ODE	220	Diphenyl Thiourea	2 to 1	1 h		27%	63%	10%
26	ODE	220	Diphenyl Thiourea	4 to 1	1 h		60%		40%
27	ODE	220	1-Hexyl-3-Phenyl Thiourea	1 to 1	1 h			77%	23%
28	ODE	270	1-Hexyl-3-Phenyl Thiourea	2 to 1	1 h		53%	18%	29%
29	ODE	270	1-Hexyl-3-Phenyl Thiourea	4 to 1	1 h		46%		54%
30	ODE	270	1-Hexyl-3-Phenyl Thiourea	6 to 1	1 h		74%		26%

31	ODE	155	Diethyl Thiourea	6 to 1	1 h			25%	75%
32	ODE	155	Diethyl Thiourea	12 to 1	1 h			8%	92%
33	ODE	170	Diethyl Thiourea	3 to 1	1 h			47%	53%
34	ODE	170	Diethyl Thiourea	6 to 1	1 h			46%	54%
35	ODE	170	Diethyl Thiourea	9 to 1	1 h			42%	58%
36	ODE	170	Diethyl Thiourea	12 to 1	1 h		51%		49%
37	ODE	170	Diethyl Thiourea	9 to 1	10 min			37%	63%
38	ODE	170	Diethyl Thiourea	9 to 1	30 min			50%	50%
39	ODE	155	Diethyl Thiourea Followed by 5 mL of oleic acid after reaction	18 to 1	1 hour				100%
18	ODE	220	1-Hexyl-3-Phenyl Thiourea	0.5 to 1	20 min			100%	

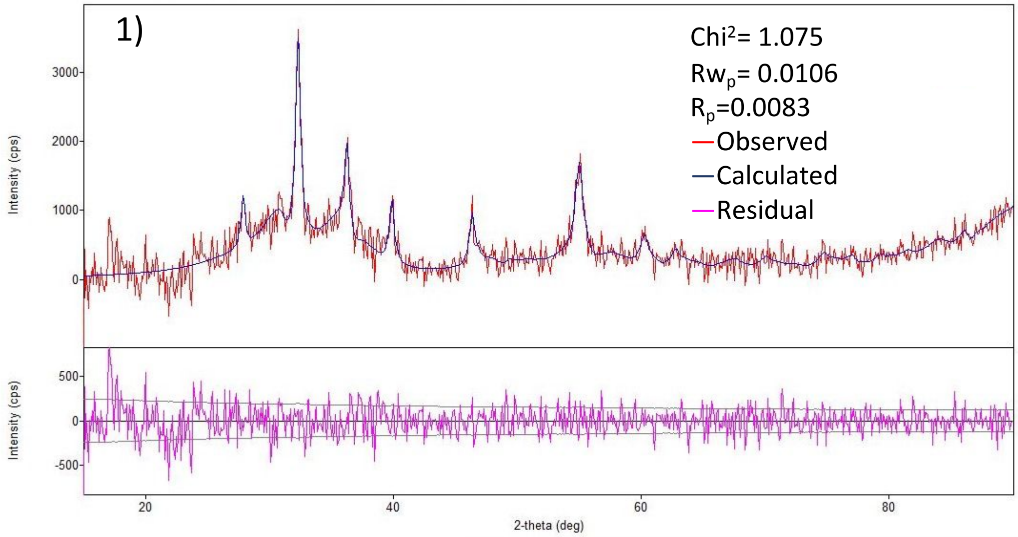
1)
Chi²= 1.075
Rwp= 0.0106
Rp=0.0083
Observed
Calculated
Residual
Intensity (cps)
3000
2000
1000
0
500
0
-500
20
40
60
80
2-theta (deg)

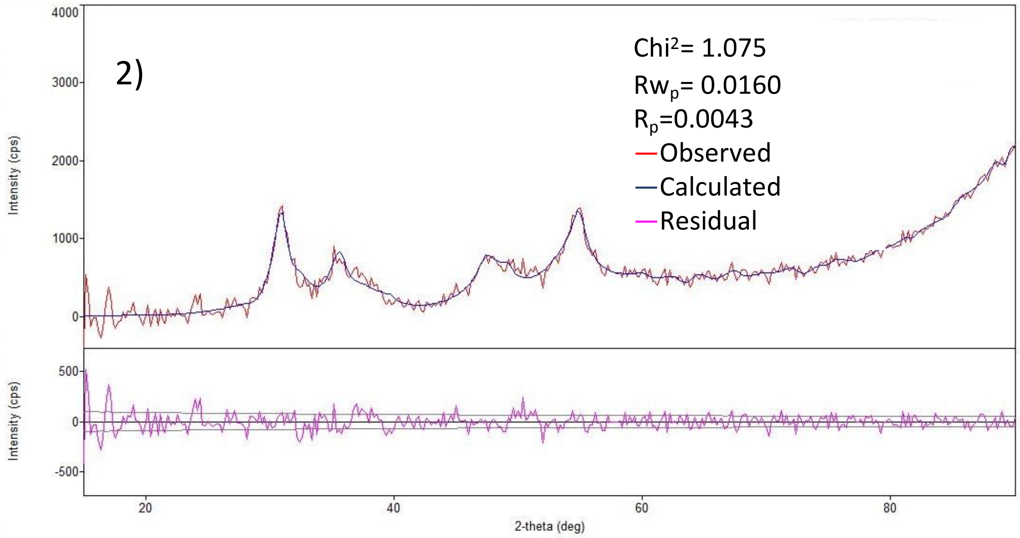
2)
Chi²= 1.075
Rwp= 0.0160
Rp=0.0043
Observed
Calculated
Residual
Intensity (cps)
4000
3000
2000
1000
0
500
0
-500
20
40
60
80
2-theta (deg)

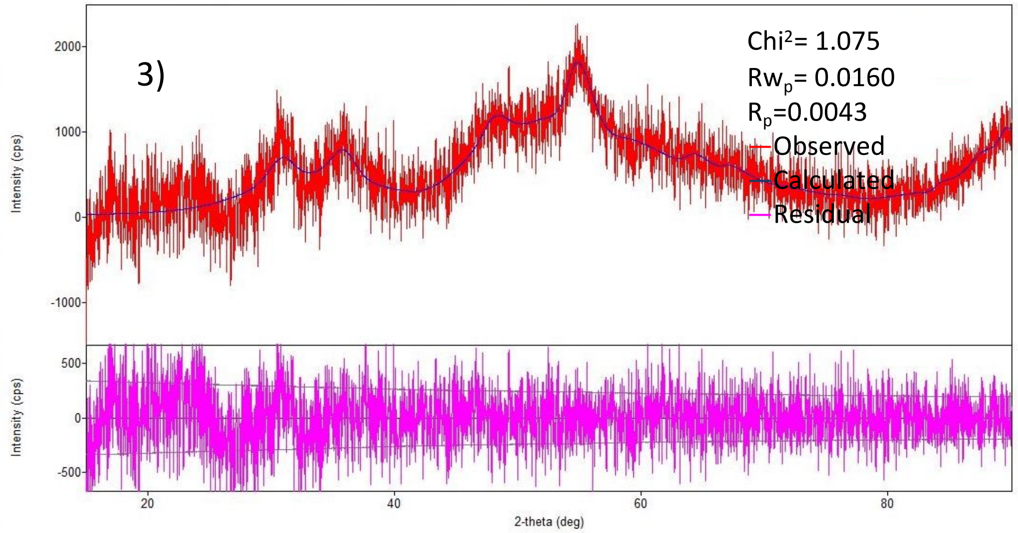
3)
Chi²= 1.075
Rwp= 0.0160
Rp=0.0043
Observed
Calculated
Residual
Intensity (cps)
2000
1000
0
-1000
500
0
-500
20
40
60
80
2-theta (deg)

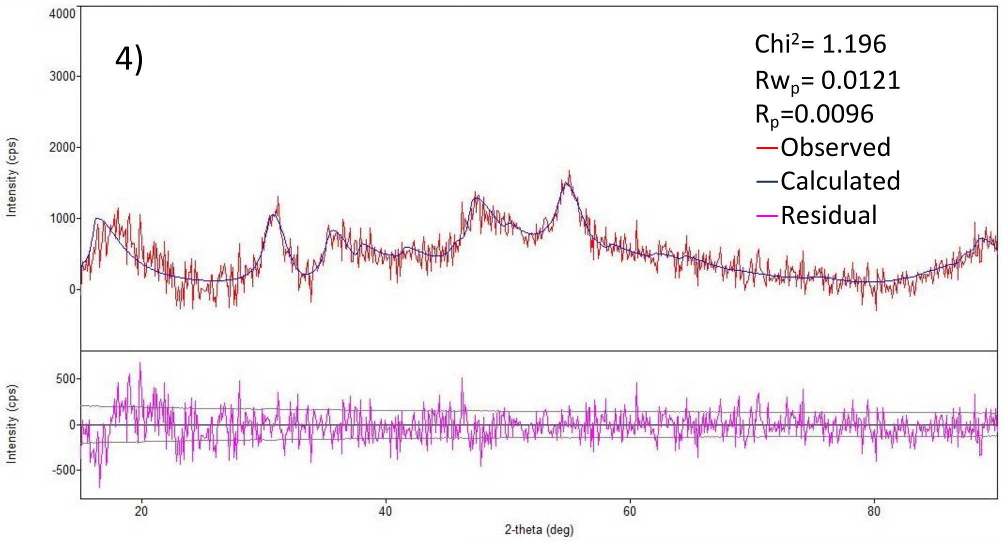
4)
Chi²= 1.196
Rwp= 0.0121
Rp=0.0096
Observed
Calculated
Residual
Intensity (cps)
2-theta (deg)

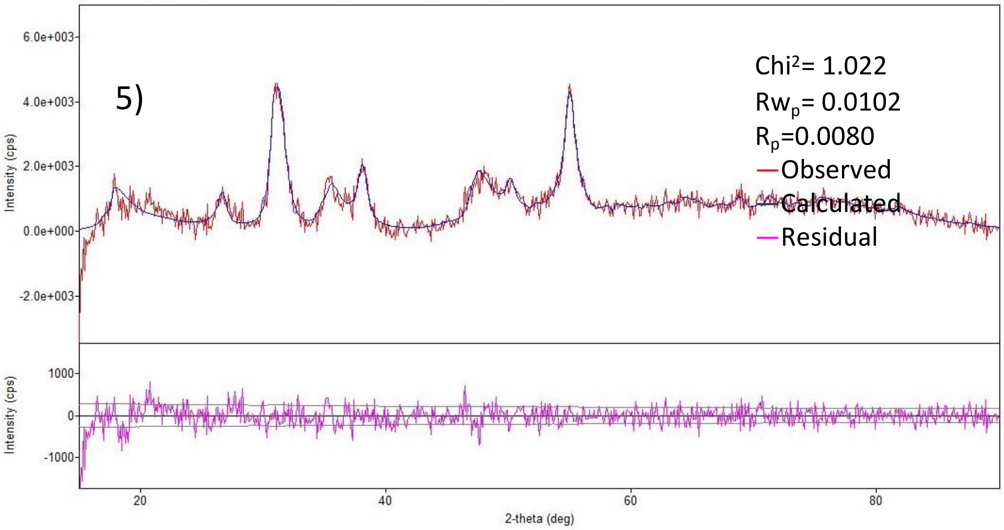
5)
Chi²= 1.022
Rwp= 0.0102
Rp=0.0080
Observed
Calculated
Residual
Intensity (cps)
2-theta (deg)

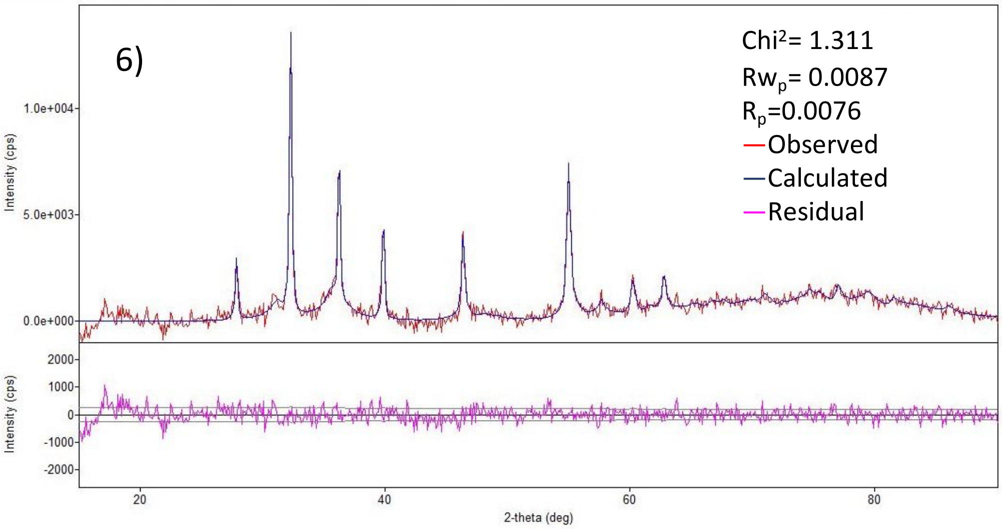
6)
Chi²= 1.311
Rwp= 0.0087
Rp=0.0076
Observed
Calculated
Residual
Intensity (cps)
2-theta (deg)

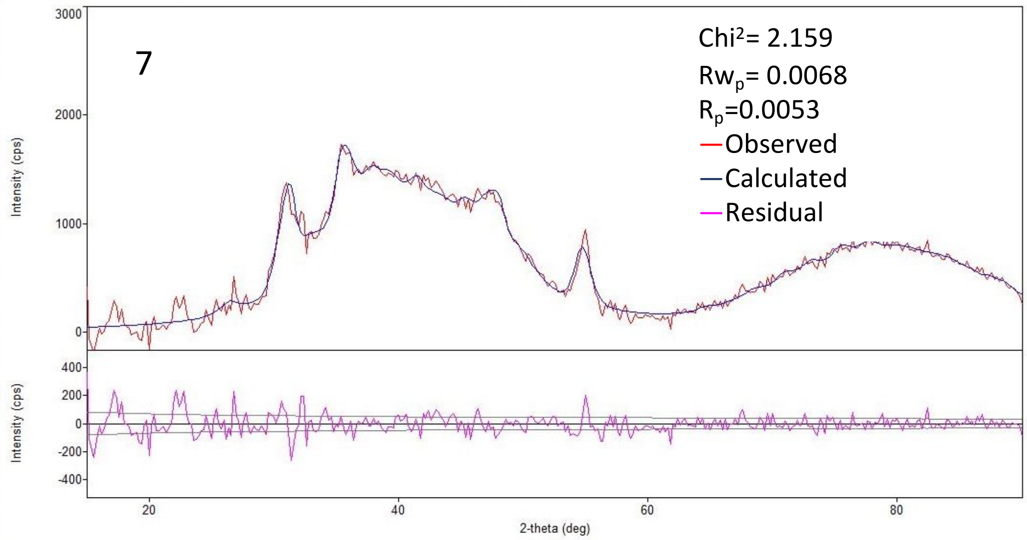
7
Chi2= 2.159
Rwp= 0.0068
Rp=0.0053
Observed
Calculated
Residual
Intensity (cps)
2-theta (deg)

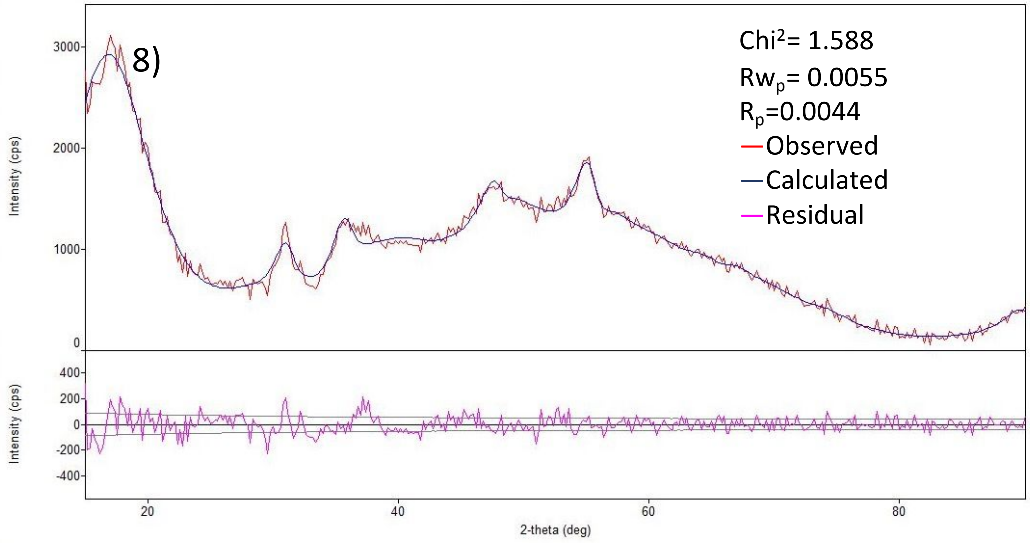
8)
Chi2= 1.588
Rwp= 0.0055
Rp=0.0044
Observed
Calculated
Residual
Intensity (cps)
2-theta (deg)

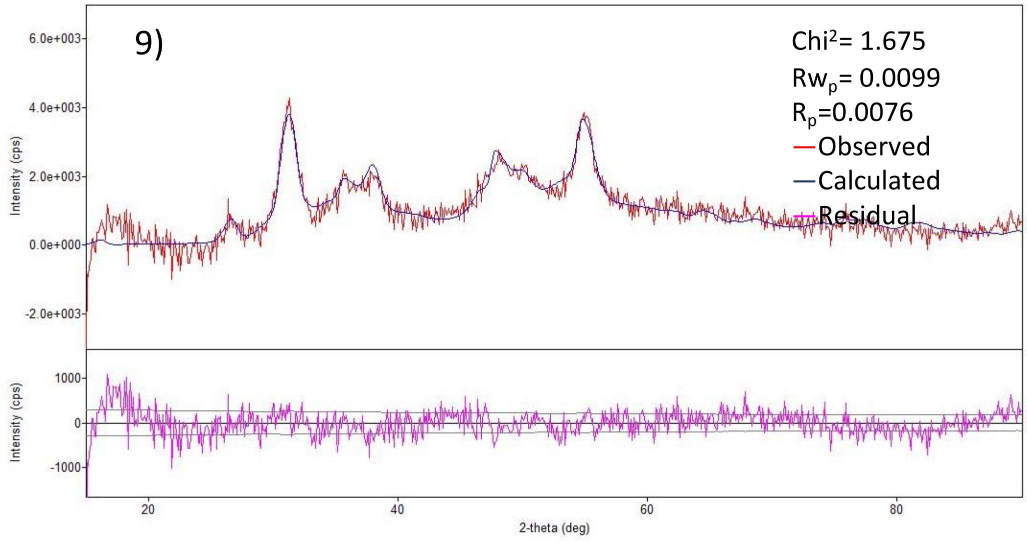
9)
Chi2= 1.675
Rwp= 0.0099
Rp=0.0076
Observed
Calculated
Residual
Intensity (cps)
2-theta (deg)

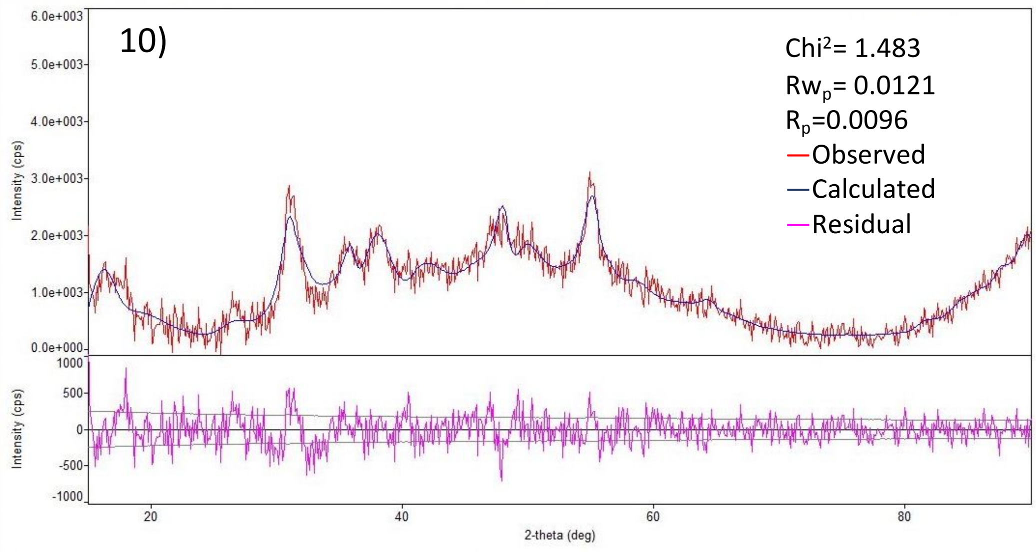
10)
Chi²= 1.483
Rwp= 0.0121
Rp=0.0096
Observed
Calculated
Residual
Intensity (cps)
2-theta (deg)

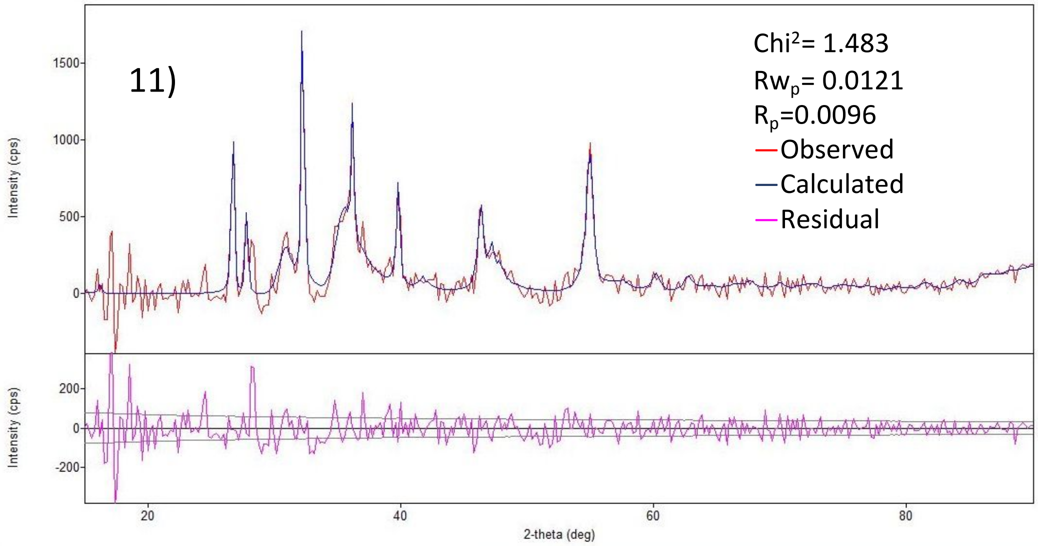
11)
Chi²= 1.483
Rwp= 0.0121
Rp=0.0096
Observed
Calculated
Residual
Intensity (cps)
2-theta (deg)

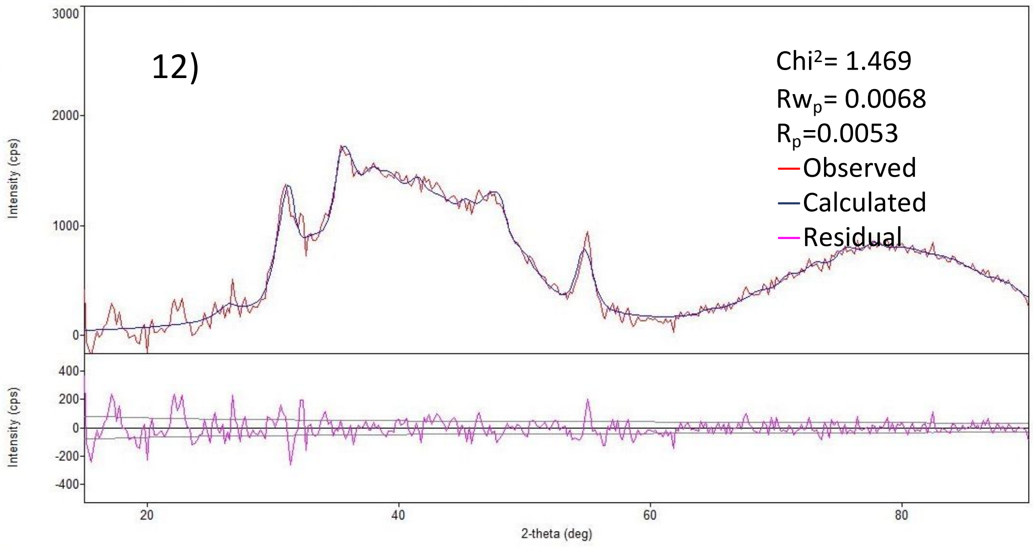
12)
Chi²= 1.469
Rwp= 0.0068
Rp=0.0053
Observed
Calculated
Residual
Intensity (cps)
2-theta (deg)

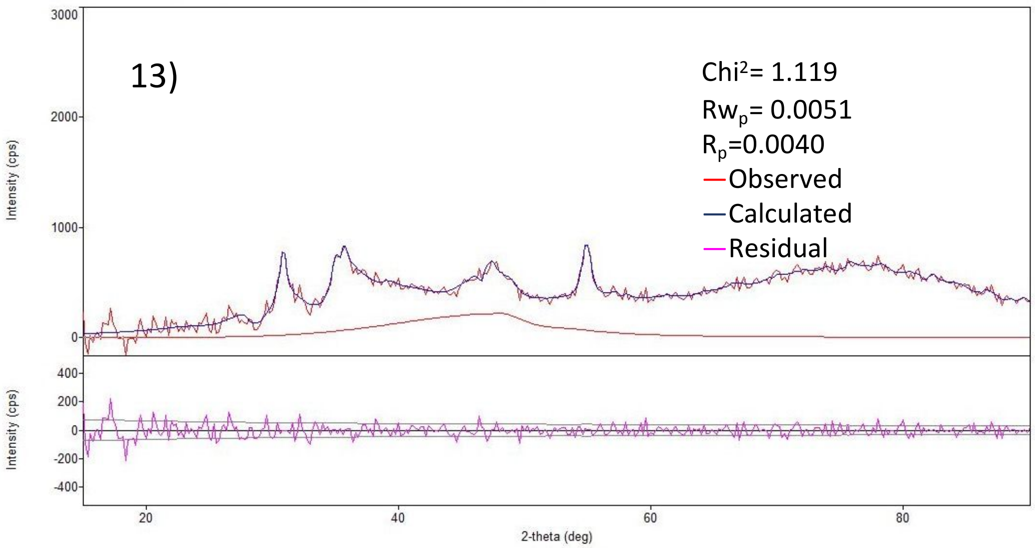
13)
Chi²= 1.119
Rwp= 0.0051
Rp=0.0040
Observed
Calculated
Residual
Intensity (cps)
2-theta (deg)

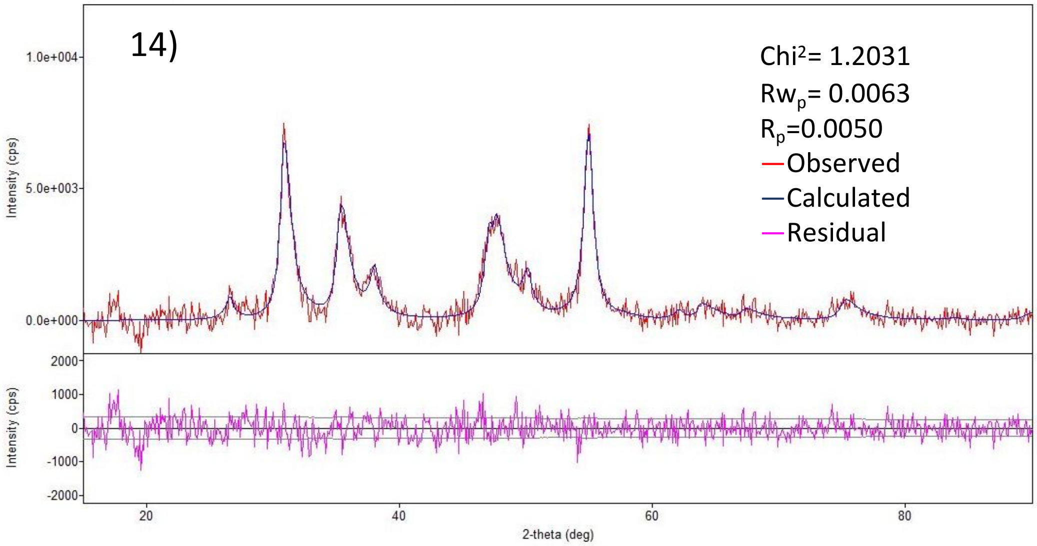
14)
Chi²= 1.2031
Rwp= 0.0063
Rp=0.0050
Observed
Calculated
Residual
Intensity (cps)
2-theta (deg)

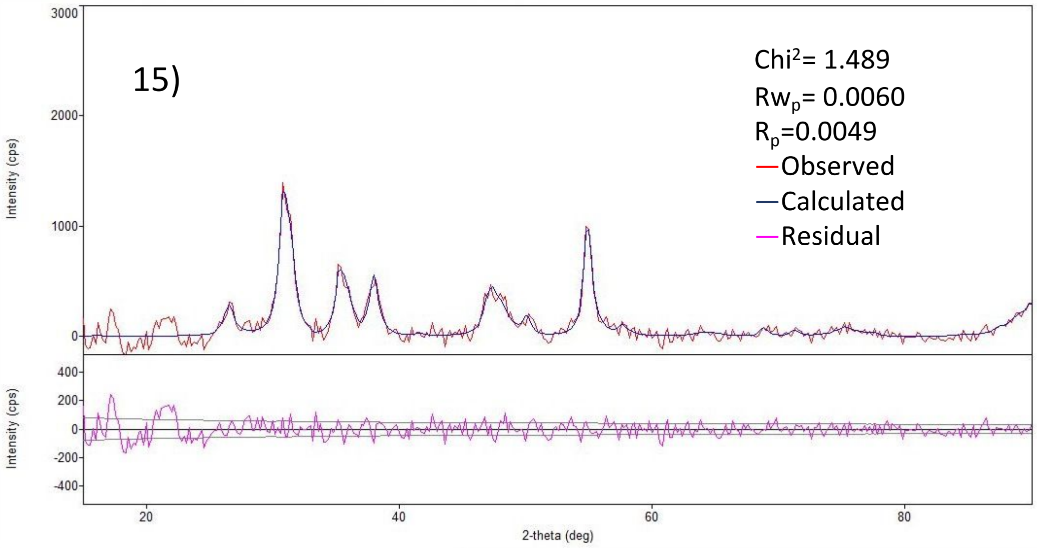
15)
Chi²= 1.489
Rwp= 0.0060
Rp=0.0049
Observed
Calculated
Residual
Intensity (cps)
2-theta (deg)

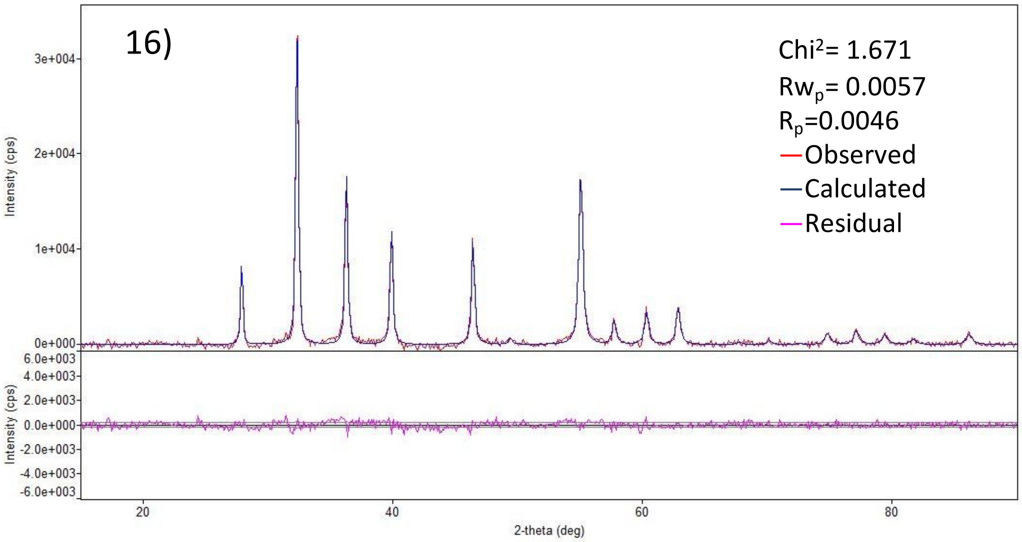
16)
Chi²= 1.671
Rwp= 0.0057
Rp=0.0046
Observed
Calculated
Residual
Intensity (cps)
2-theta (deg)

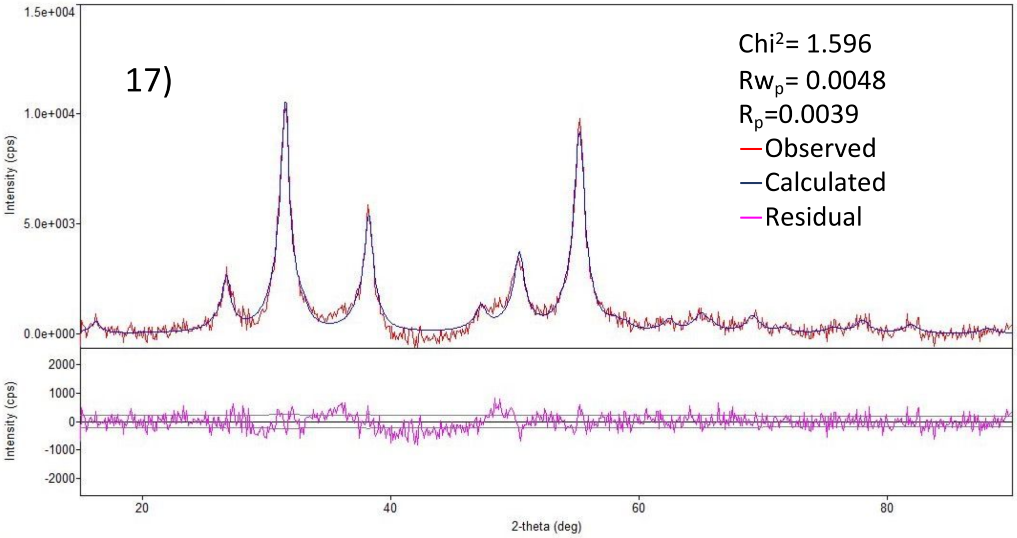
17)
Chi²= 1.596
Rwp= 0.0048
Rp=0.0039
Observed
Calculated
Residual
Intensity (cps)
2-theta (deg)

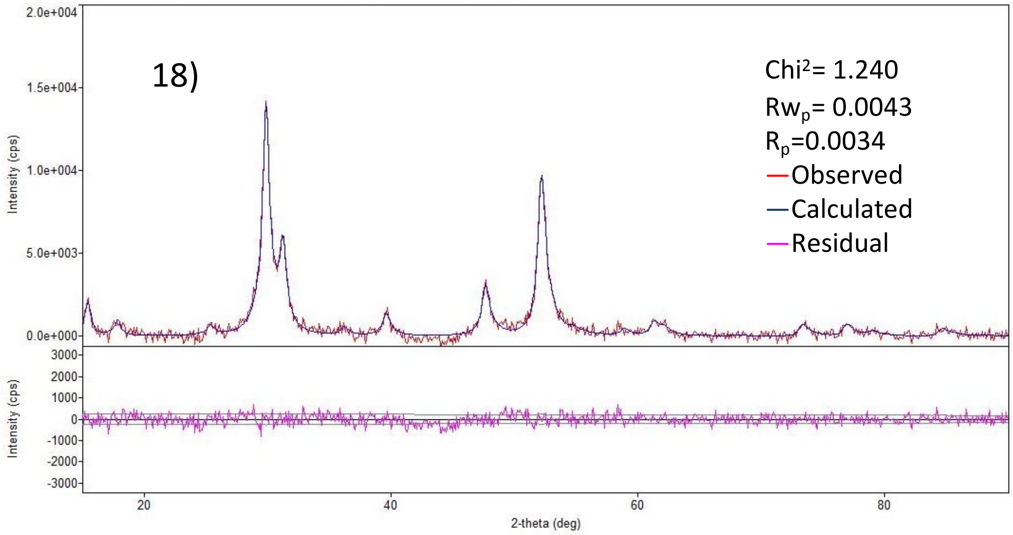
18)
Chi²= 1.240
Rwp= 0.0043
Rp=0.0034
Observed
Calculated
Residual
Intensity (cps)
2-theta (deg)

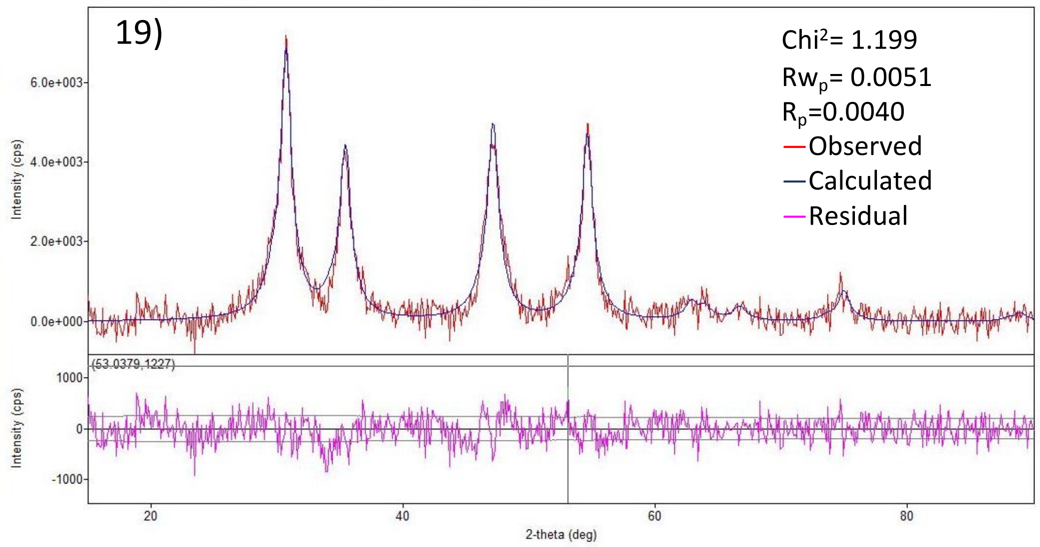
19)
Chi2= 1.199
Rwp= 0.0051
Rp=0.0040
Observed
Calculated
Residual
Intensity (cps)
6.0e+003
4.0e+003
2.0e+003
0.0e+000
(53.0379,1227)
1000
0
-1000
20
40
60
80
2-theta (deg)

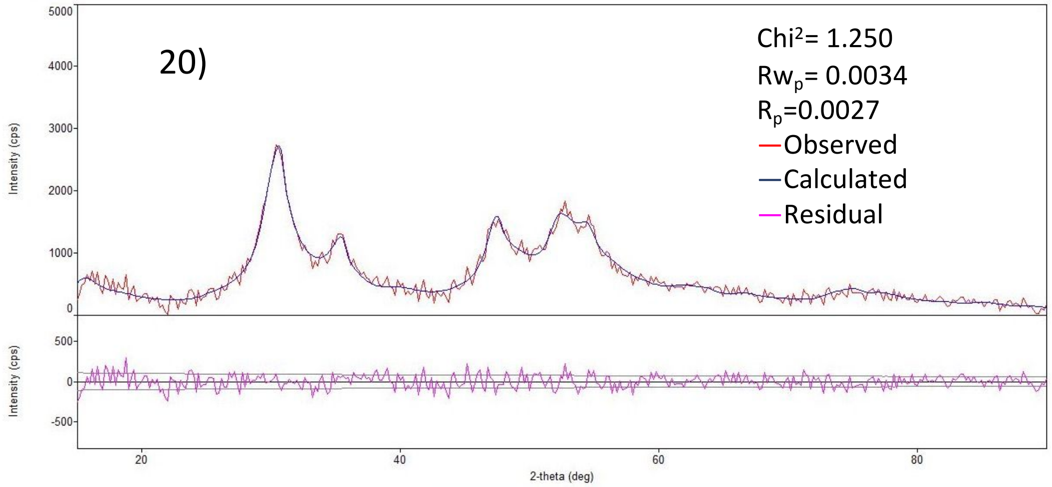
20)
Chi2= 1.250
Rwp= 0.0034
Rp=0.0027
Observed
Calculated
Residual
Intensity (cps)
5000
4000
3000
2000
1000
0
500
0
-500
20
40
60
80
2-theta (deg)

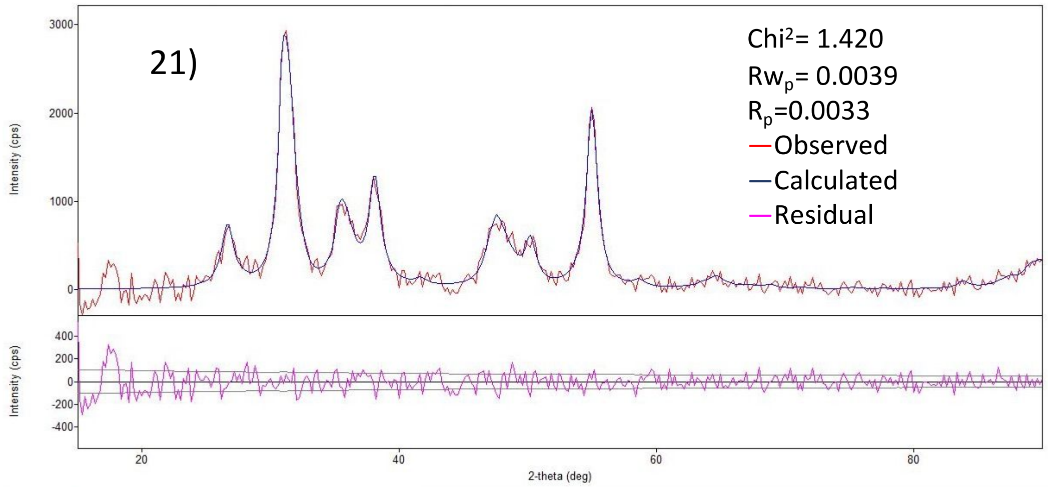
21)
Chi2= 1.420
Rwp= 0.0039
Rp=0.0033
Observed
Calculated
Residual
Intensity (cps)
3000
2000
1000
0
400
200
0
-200
-400
20
40
60
80
2-theta (deg)

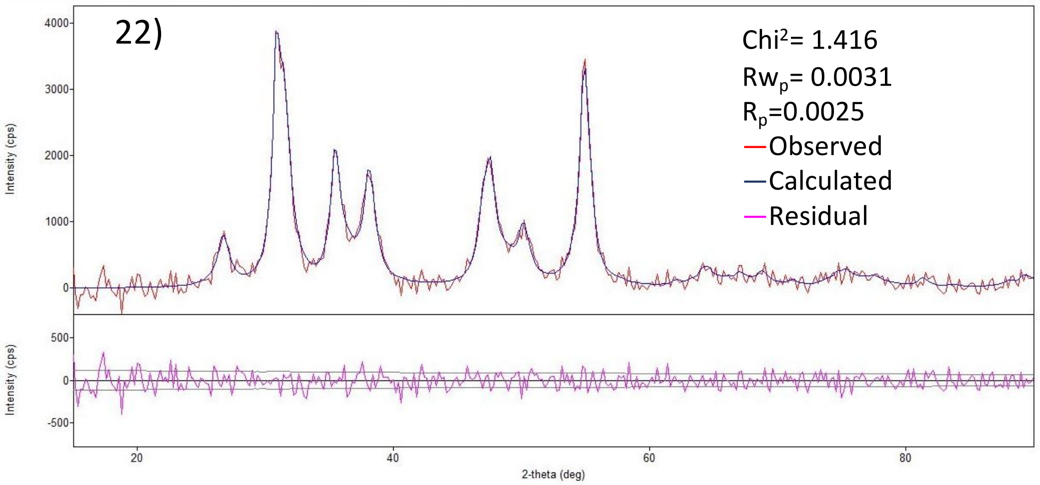
22)
Chi^2= 1.416
Rw_p= 0.0031
R_p=0.0025
Observed
Calculated
Residual
Intensity (cps)
2-theta (deg)

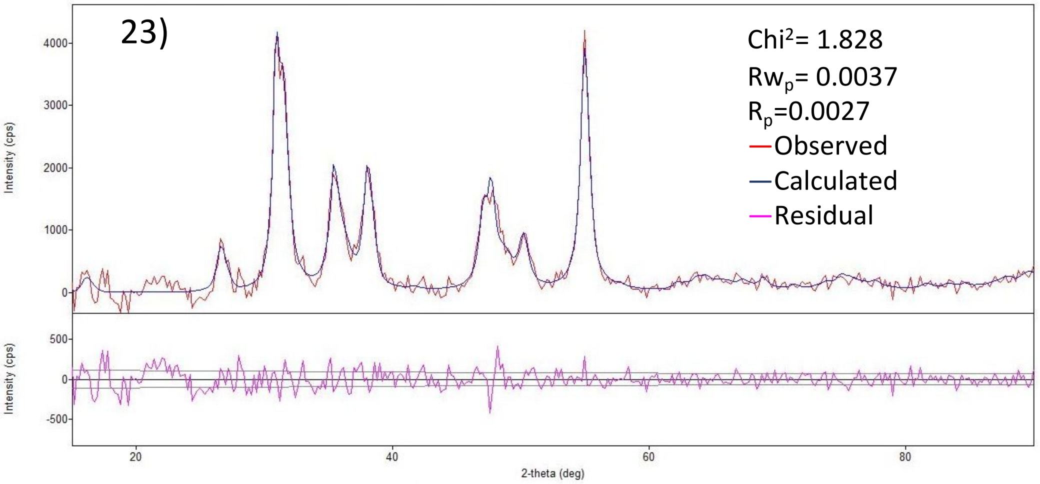
23)
Chi^2= 1.828
Rw_p= 0.0037
R_p=0.0027
Observed
Calculated
Residual
Intensity (cps)
2-theta (deg)

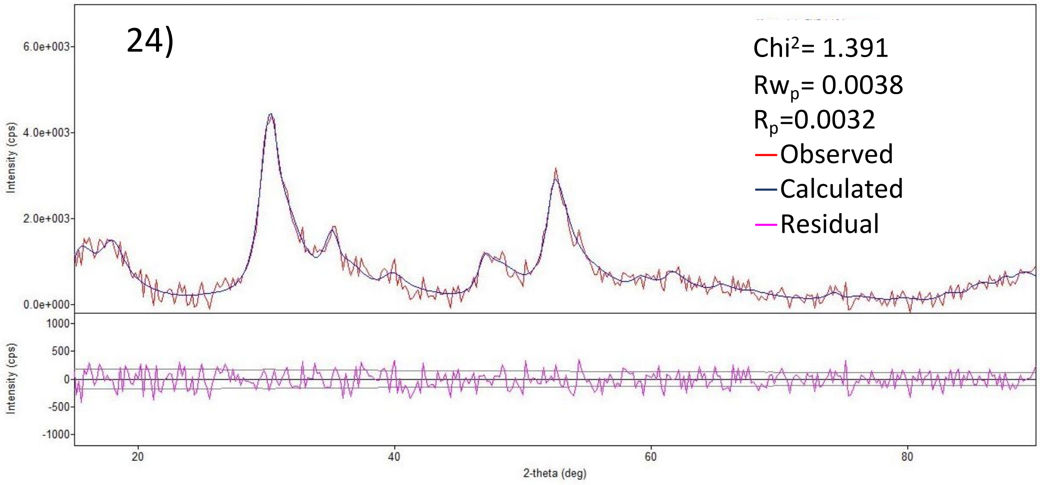
24)
Chi^2= 1.391
Rw_p= 0.0038
R_p=0.0032
Observed
Calculated
Residual
Intensity (cps)
2-theta (deg)

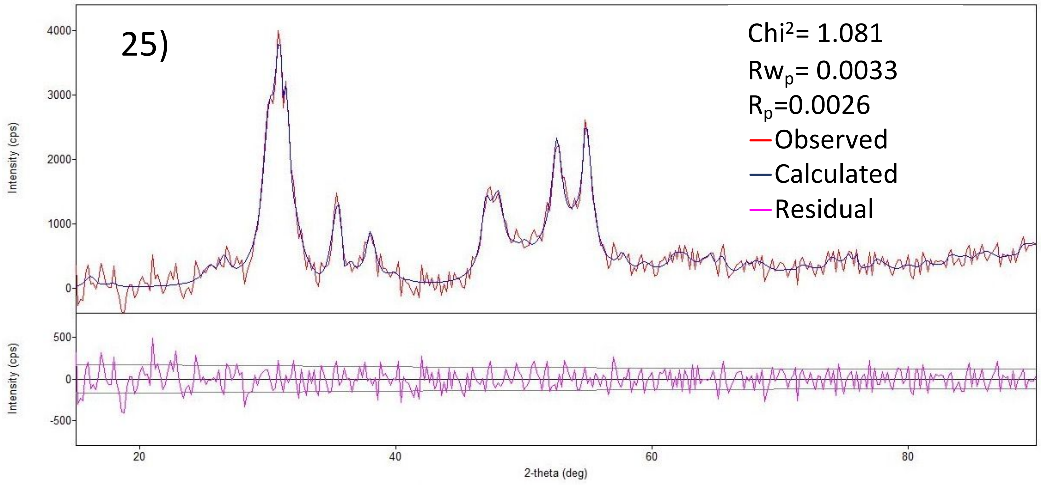
25)
Chi2= 1.081
Rwp= 0.0033
Rp=0.0026
Observed
Calculated
Residual
Intensity (cps)
2-theta (deg)

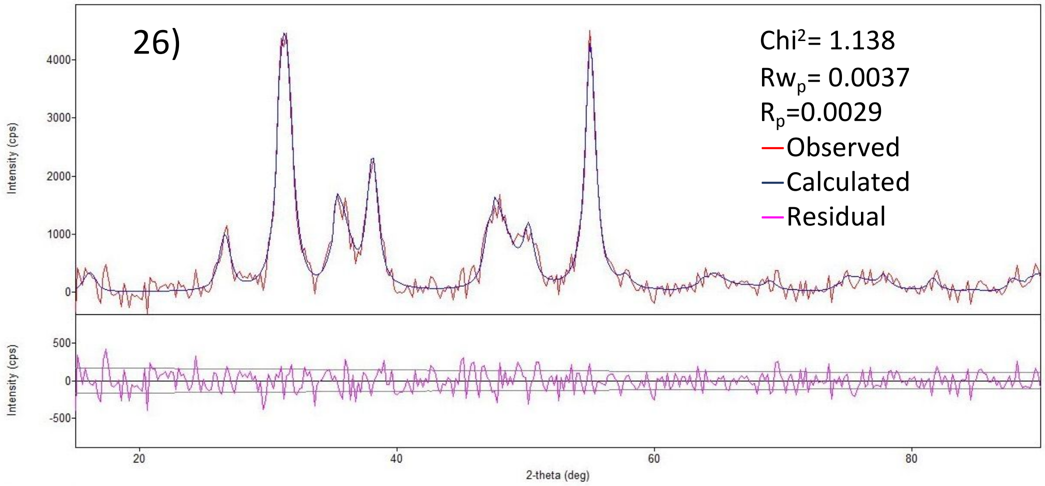
26)
Chi2= 1.138
Rwp= 0.0037
Rp=0.0029
Observed
Calculated
Residual
Intensity (cps)
2-theta (deg)

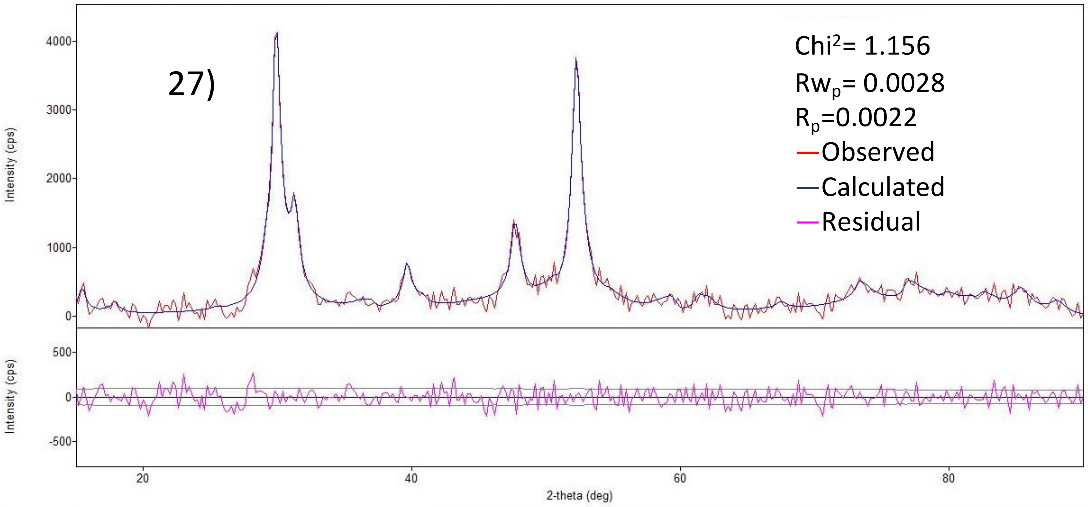
27)
Chi2= 1.156
Rwp= 0.0028
Rp=0.0022
Observed
Calculated
Residual
Intensity (cps)
2-theta (deg)

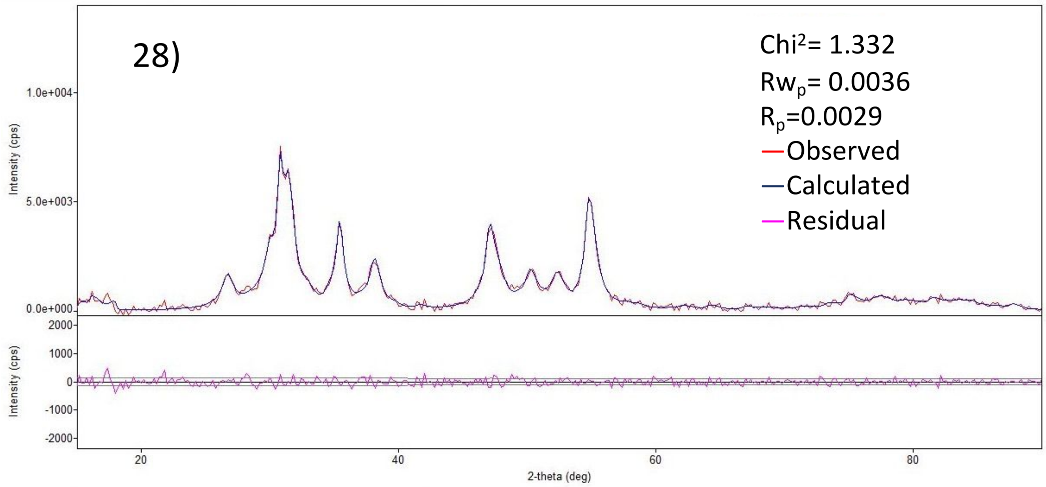
28)
Chi²= 1.332
Rwp= 0.0036
Rp=0.0029
Observed
Calculated
Residual
1.0e+004
5.0e+003
0.0e+000
Intensity (cps)
2000
1000
0
-1000
-2000
20
40
60
80
2-theta (deg)

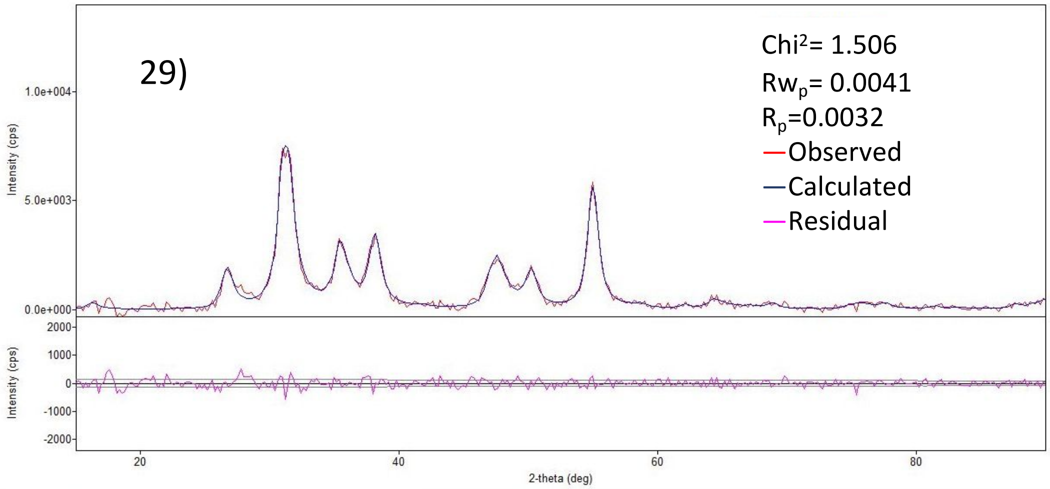
29)
Chi²= 1.506
Rwp= 0.0041
Rp=0.0032
Observed
Calculated
Residual
1.0e+004
5.0e+003
0.0e+000
Intensity (cps)
2000
1000
0
-1000
-2000
20
40
60
80
2-theta (deg)

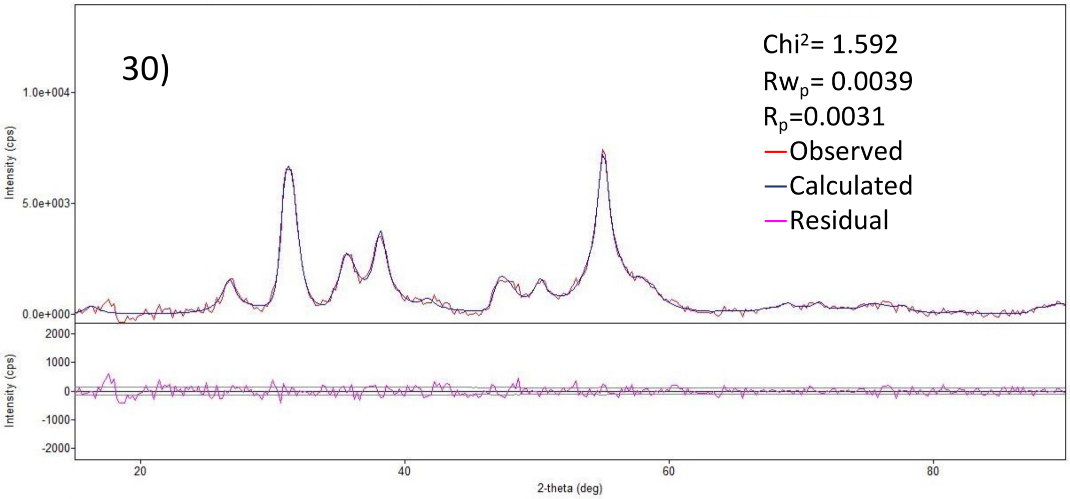
30)
Chi²= 1.592
Rwp= 0.0039
Rp=0.0031
Observed
Calculated
Residual
1.0e+004
5.0e+003
0.0e+000
Intensity (cps)
2000
1000
0
-1000
-2000
20
40
60
80
2-theta (deg)

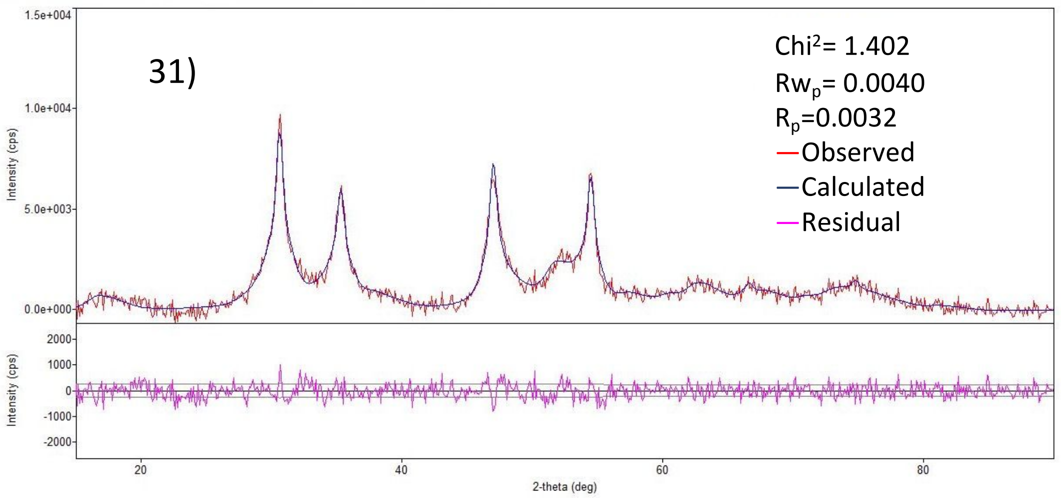
31)
Chi2= 1.402
Rwp= 0.0040
Rp=0.0032
—Observed
—Calculated
—Residual
Intensity (cps)
1.5e+004
1.0e+004
5.0e+003
0.0e+000
2000
1000
0
-1000
-2000
20
40
60
80
2-theta (deg)

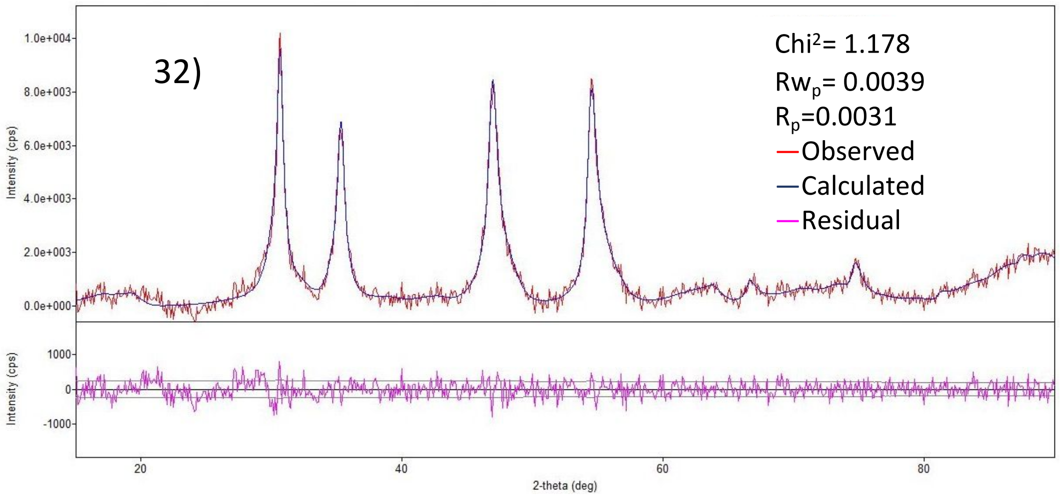
32)
Chi2= 1.178
Rwp= 0.0039
Rp=0.0031
—Observed
—Calculated
—Residual
Intensity (cps)
1.0e+004
8.0e+003
6.0e+003
4.0e+003
2.0e+003
0.0e+000
1000
0
-1000
20
40
60
80
2-theta (deg)

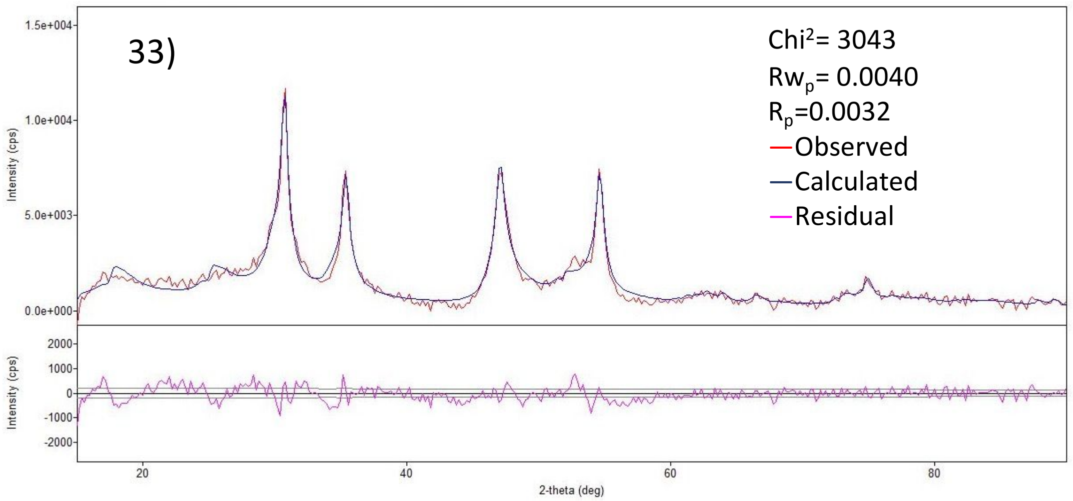
33)
Chi2= 3043
Rwp= 0.0040
Rp=0.0032
—Observed
—Calculated
—Residual
Intensity (cps)
1.5e+004
1.0e+004
5.0e+003
0.0e+000
2000
1000
0
-1000
-2000
20
40
60
80
2-theta (deg)

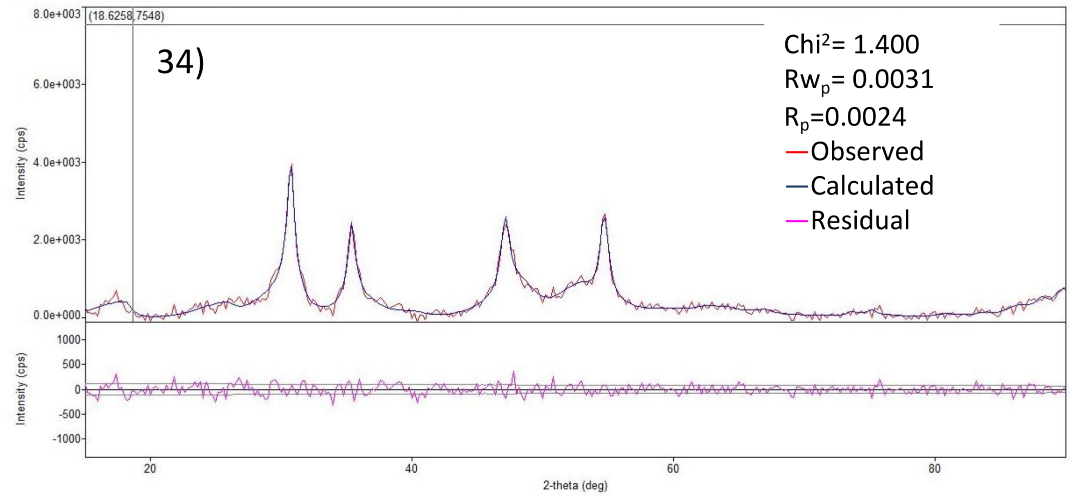

34)
(18.6258,7548)
Chi²= 1.400
Rwp= 0.0031
Rp=0.0024
Observed
Calculated
Residual
Intensity (cps)
2-theta (deg)

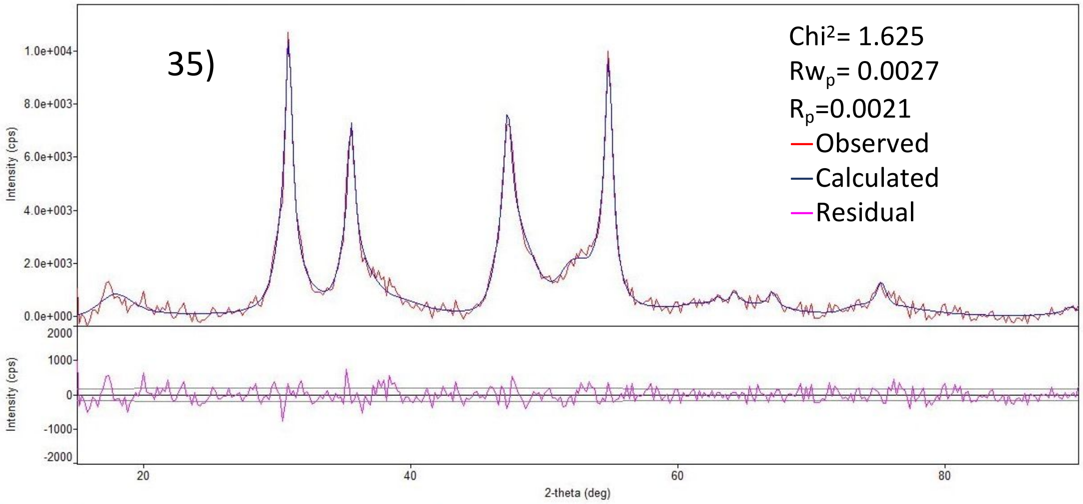

35)
Chi²= 1.625
Rwp= 0.0027
Rp=0.0021
Observed
Calculated
Residual
Intensity (cps)
2-theta (deg)

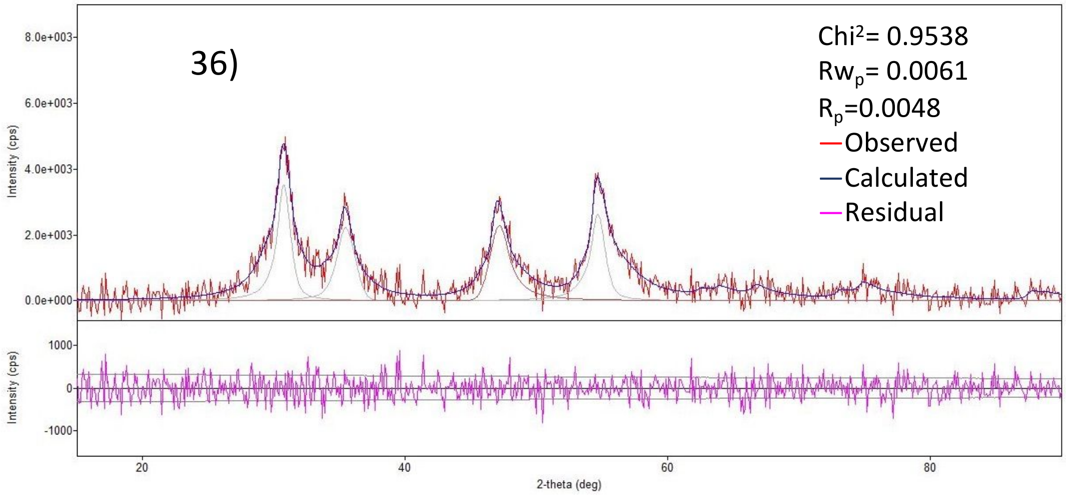

36)
Chi²= 0.9538
Rwp= 0.0061
Rp=0.0048
Observed
Calculated
Residual
Intensity (cps)
2-theta (deg)

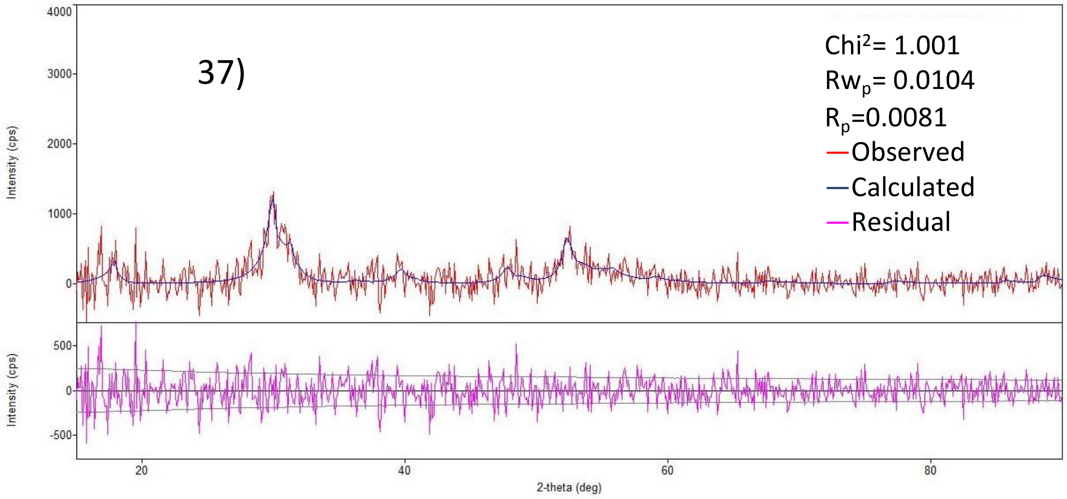

Figure S6: Rietveld refinements of the cobalt sulfides from table **Table B.1**, included in each is the experimental (red) and calculated curves (blue) as well as the difference curves (pink)
